# Endocrine disorders and fertility and pregnancy: An update

**DOI:** 10.3389/fendo.2022.970439

**Published:** 2023-01-17

**Authors:** Anna Bendarska-Czerwińska, Nikola Zmarzły, Emilia Morawiec, Agata Panfil, Kamil Bryś, Justyna Czarniecka, Aleksander Ostenda, Konrad Dziobek, Dorota Sagan, Dariusz Boroń, Piotr Michalski, Veronica Pallazo-Michalska, Beniamin Oskar Grabarek

**Affiliations:** ^1^ Department of Molecular, Biology Gyncentrum Fertility Clinic, Katowice, Poland; ^2^ Faculty of Medicine, Academy of Silesia, Zabrze, Poland; ^3^ American Medical Clinic, Katowice, Poland; ^4^ Department of Histology, Cytophysiology and Embryology, Faculty of Medicine, University of Technology, Academy of Silesia in Katowice, Zabrze, Poland; ^5^ Department of Microbiology, Faculty of Medicine, University of Technology, Academy of Silesia in Katowice, Zabrze, Poland; ^6^ Medical Center Dormed Medical SPA, Busko-Zdroj, Poland; ^7^ Department of Gynaecology and Obstetrics, Faculty of Medicine, Academy of Silesia, Zabrze, Poland; ^8^ Department of Gynecology and Obstetrics with Gynecologic Oncology, Ludwik Rydygier Memorial Specialized Hospital, Kraków, Poland; ^9^ Department of Gynecology and Obstetrics, TOMMED Specjalisci od Zdrowia, Katowice, Poland

**Keywords:** endocrine disorders, fertility, infertility, pregnancy, reproductive health, hormones

## Abstract

It is estimated that more and more couples suffer from fertility and pregnancy maintenance disorders. It is associated with impaired androgen secretion, which is influenced by many factors, ranging from genetic to environmental. It is also important to remember that fertility disorders can also result from abnormal anatomy of the reproductive male and female organ (congenital uterine anomalies – septate, unicornuate, bicornuate uterus; acquired defects of the uterus structure – fibroids, polyps, hypertrophy), disturbed hormonal cycle and obstruction of the fallopian tubes resulting from the presence of adhesions due to inflammation, endometriosis, and surgery, abnormal rhythm of menstrual bleeding, the abnormal concentration of hormones. There are many relationships between the endocrine organs, leading to a chain reaction when one of them fails to function properly. Conditions in which the immune system is involved, including infections and autoimmune diseases, also affect fertility. The form of treatment depends on infertility duration and the patient’s age. It includes ovulation stimulation with clomiphene citrate or gonadotropins, metformin use, and weight loss interventions. Since so many different factors affect fertility, it is important to correctly diagnose what is causing the problem and to modify the treatment regimen if necessary. This review describes disturbances in the hormone secretion of individual endocrine organs in the context of fertility and the maintenance of pregnancy.

## 1 Introduction

Infertility is the inability to conceive within 12 months of regular intercourse (2-4 times a week) without using any contraceptive methods. Involuntary childlessness is a significant social problem faced by 20% of couples worldwide, and only in Poland the problem of infertility affects approximately 1.5 million couples each year (1).

Factors that influence fertility in both sexes include hyperprolactinemia, hypogonadotropic hypogonadism, infections, systemic diseases, and even lifestyle (2). More and more attention is also paid to the problem of obesity. Adipocytes act as an endocrine organ, and their excess promotes disorders of the hypothalamic-pituitary-ovarian axis. Secreted adipokines include leptin, adiponectin, resistin, interleukin 6, interleukin 1β, and tumor necrosis factor α (TNFα), involved in inflammatory processes and the regulation of metabolism (3).

According to the recommendations of the Fertility and Infertility Section at the Polish Society of Gynecologists and Obstetricians and the Polish Society of Reproductive Medicine and Embryology, there are several causes of male infertility (4): 1) pre-testicular, i.e. related to the malfunctioning of the endocrine system in terms of impaired secretion of luteinizing hormone, sex steroids – testosterone, inhibin, folliculostimulin or resulting from mutations which cause impaired sperm movement or chromosomal aberrations observed, e.g. in Klinefelter’s syndrome, 2) testicular, e.g. cryptorchidism, varicocele, infectious diseases; 3) extra-testicular, which include congenital absence of vas deferens, underdevelopment of seminal vesicles, epididymis defects, polycystic kidney disease, cystic fibrosis, diabetes; 4) sexual disorders – lack of erection or ejaculation, penis structure abnormalities (5). During the diagnosis of infertility, the assessment of male fertility should be obligatory, and the examination should be performed after maintaining a 2-7 day period of sexual abstinence (6).

On the other hand, infertility in women may be mainly due to: disturbances in the occurrence of ovulatory cycles, caused, inter alia, by woman’s age, hormonal disorders in the course of polycystic ovary syndrome (PCOS), hyperprolactinemia, abnormal anatomy of the reproductive organ (congenital uterine anomalies – septate, unicornuate, bicornuate uterus; acquired defects of the uterus structure – fibroids, polyps, hypertrophy), disturbed hormonal cycle and obstruction of the fallopian tubes resulting from the presence of adhesions due to inflammation, endometriosis, and surgery (7).

Diagnostics of female infertility should include: medical history regarding the regularity of menstruation, gynecological examination, determination of the concentration of selected sex hormones, including a single measurement of progesterone level 7 days before the planned menstruation to assess ovulation, ultrasound (USG) at the end of the follicular phase of the cycle (8, 9). However, in the case of ovulation disorders, manifested by an abnormal rhythm of menstrual bleeding, progesterone level lower than 2 ng/mL in the middle of the luteal phase, the diagnosis should be supplemented with the determination of the concentration of gonadotropins, androgens, thyroid-stimulating hormone (TSH), prolactin (PRL), anti-müllerian hormone (AMH) and assessment of the reproductive potential of the ovaries (10). The form of infertility treatment depends on its duration and the patient’s age. It includes ovulation stimulation with clomiphene citrate or gonadotropins, intrauterine insemination, *in vitro* fertilization (11).

**Figure 1:**
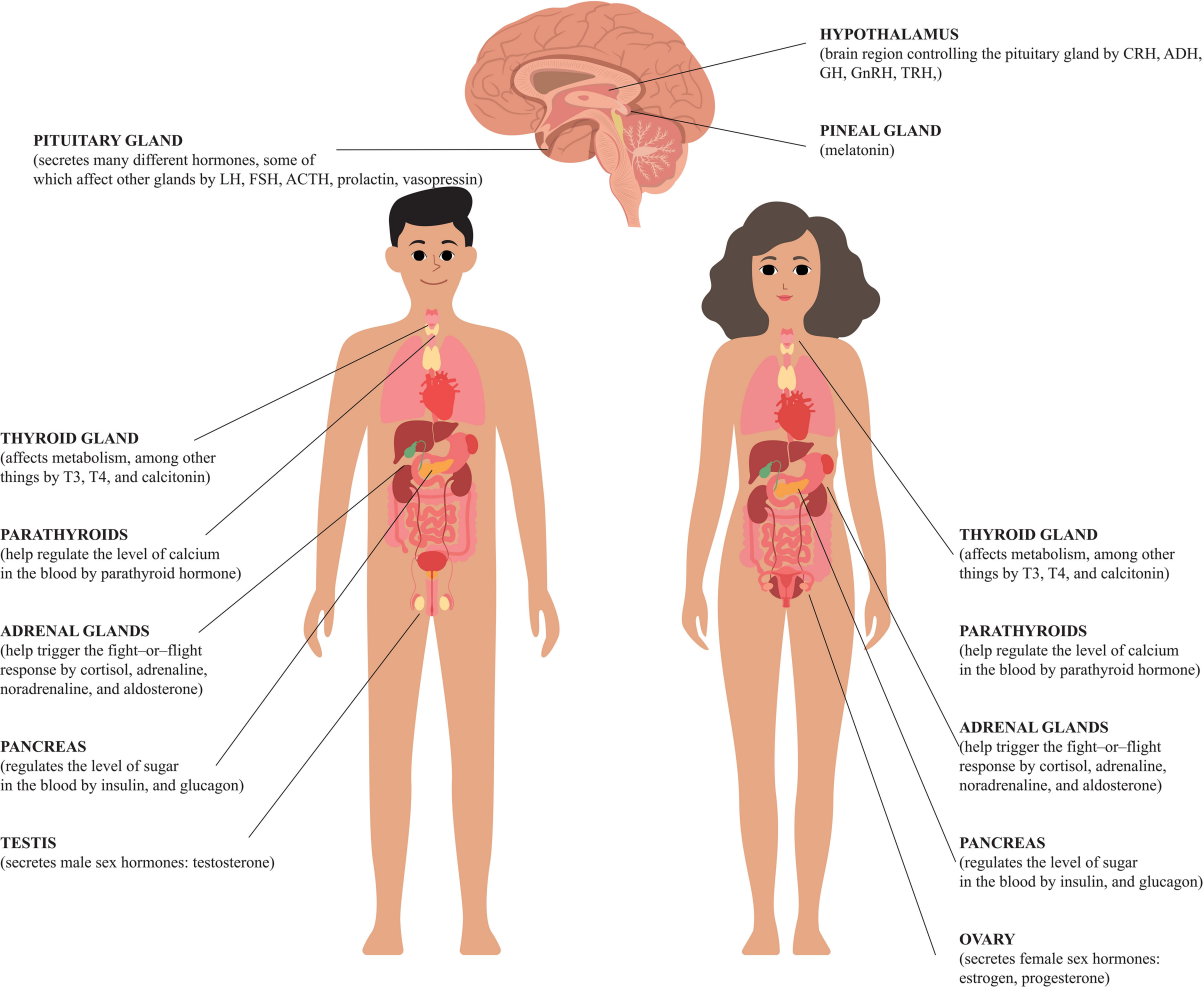
Human hormones and their roles.

The aim of this review was to present disturbances in the hormone secretion of individual endocrine organs in the context of fertility of both sexes and the maintenance of pregnancy (**Figure 1**).

## 2 Differences between male and female embryogenesis

Sex differentiation is a complex process that depends on the activity of many genes. The key to sex dimorphism is the SRY (sex-determining region on Y) gene, whose protein product initiates the cascade of expression of genes determining the formation of testicles. The lack of this protein allows development towards a female individual (12, 13).

### 2.1 Undifferentiated stage

The sex of the embryo is determined already during fertilization, but until the 7th week of development, the gonads do not show gender-related differences. Initially, the gonads are in the form of longitudinal folds, which are formed due to the proliferation of the body cavity epithelium and the thickening of the stromal mesenchyme. Primordial germ cells appear no earlier than the 6th week of development and it is important for the gonads development that they populate the genital crests. Genital crest epithelial cells proliferate and penetrate into the stromal mesenchyme, where they form irregularly shaped primary sex cords. In both female and male embryos, these cords maintain a connection to the surface epithelium. This makes it impossible to distinguish between male and female gonads, which is why these structures are referred to as undifferentiated gonads (14).

Female and male embryos have two pairs of ducts: mesonephric (Wolffian) and paramesonephric (Müllerian). The caudal part of the connected ducts is incorporated into the posterior wall of the urogenital sinus, forming a sinus tubercle.

At 3 weeks of development, the mesenchyme cells migrate around the cloacal membrane, forming a pair of slightly elevated cloacal folds. They join cranially to form the genital tubercle, and caudally to the urethral and the anal folds. At the same time, another pair of genital eminences is visible on either side of the urethral folds. Later, they form the scrotal eminences in men and the labia majora in women (15).

### 2.2 Embryogenesis of the female reproductive system

#### 2.2.1 Ovaries

In female development, the primary sex cords break down into irregular clusters of cells. During further development, they disappear and are replaced by vascularized stroma tissue, forming the ovarian core. The surface epithelial cells of the female gonad (as opposed to the epithelial cells of the male gonad) continue to divide, resulting in the formation of cortical cords around the 7th week of development. In the third month, they disintegrate into separate cell foci forming follicular cells, creating, together with the oogonium, the primary (resting) follicle (16).

#### 2.2.2 Genital ducts

In the presence of estrogen, the main female genital ducts are formed from the paramesonephric duct. With the descent of the ovary, part of the ducts transform into the fallopian tube and uterine canal. The connected paramesonephric ducts become the corpus and cervix and the upper part of the vagina. In female embryos, the mesonephric ducts regress due to a lack of testosterone (16).

#### 2.2.3 External genitalia

The development of female external genitalia is stimulated by estrogens. The genital tubercle lengthens to form the clitoris, and the folds of the urethra give rise to the labia minora. In turn, the vaginal prominences enlarge and produce the labia majora. The urethral groove remains open and forms the vestibule (17).

### 2.3 Embryogenesis of the male reproductive system

#### 2.3.1 Testes

The genetically male embryo, under the influence of the SRY gene product, develops sex cords that penetrate deeper towards the core part of the gonad, forming medullary cords (13). These, in turn, continue to disintegrate, becoming the tubules of the testicle network. In further development, the testicular cords are separated from the surface epithelium by the tunica albuginea. In the fourth month, the testicular cords join the nucleus network. It is worth noting that the testicular cords retain the form of solid structures until puberty, and then transform into convoluted seminiferous tubules (14, 18).

#### 2.3.2 Genital ducts

They develop under the influence of testosterone and originate from part of the mesonephric organ. With the exception of the appendix of the epididymis, the mesonephric duct does not regress and forms the main genital ducts. Below the orifice of the efferent ducts, the epididymal duct is elongated and formed. The mesonephric duct from the tail of the epididymis to the protrusion of the seminal vesicle is called the vas deferens.

#### 2.3.3 External genitalia

The development of male external genitalia is stimulated by androgens and is manifested by the rapid lengthening of the genital tubercle, the penis. In the process of elongation, the penis pulls on the folds of the urethra, which form the urethral grooves. The furthest part is the penis glans. The epithelial lining of the groove forms the urethral plate. At the end of the 3rd month, the folds of the urethra close above the plate, forming the spongy part of the urethra, then the urethral lumen is formed. In turn, the genital (scrotal) eminences move in the caudal direction during development and form the scrotum (17).

## 3 Hypothalamus and pituitary gland

The hypothalamus and pituitary gland control and regulate the proper functioning of the endocrine glands. The gonadotropic pathway is especially important in the case of fertility and the maintenance of pregnancy (**Tables 1**, **2**). The hypothalamus produces gonadotropin-releasing hormone (GnRH), the appropriate concentration of which is necessary for the secretion of gonadotropins, i.e. luteinizing hormone (LH) and follicle-stimulating hormone (FSH) by the anterior (glandular) lobe of the pituitary gland (22). Gonadotropins, in turn, have a direct influence on the production of sex hormones, sperm and ova, as well as the course of pregnancy. Thus, the malfunction of the hypothalamus or pituitary gland will be associated with low production of GnRH and gonadotropins, which in turn will lead to the gonadal failure, known as hypogonadotropic hypogonadism (23).

**Table 1:** Fertility studies related to the hypothalamus and pituitary gland.

Authors (reference)	Type of study	Study design	Aim	Results	Conclusion
Abbara et al. (19)	*in vivo* study (human) female	Comparative study	Evaluation of gonadotropin levels in a group of 243 patients with hyperprolactinemia	In patients with PCOS and hyperprolactinemia, there is an increase mainly in LH	Depending on the severity of hyperprolactinemia, a variable pattern of gonadotropin secretion is noted
Feng et al. (20)	*in vivo* study (human) female	Comparative study	Evaluation of the effectiveness of the use of Bushen-zhu-yun decoction (BSZY-D) and dopamine agonists in infertile patients with hyperprolactinemia	Adding decoction to traditional treatment results in a reduction in the number of miscarriages, fewer side effects, lower prolactin levels	Bushen-zhu-yun decoction (BSZY-D) and dopamine agonists are characterized by synergism of action, which is beneficial
Sermondade et al. (21)	*in vivo* study (human) male	Meta-analysis	Confirmation of the relationship between BMI and sperm count	Overweight and obesity were associated with an increased prevalence of azoospermia or oligozoospermia	BMI is associated with sperm count

**Table 2:** Fertility studies related to the pineal gland activity.

Authors (reference)	Type of study	Study design	Aim	Results	Conclusion
Espino et al. (25)	*in vivo* human female	Clinical experiment	Assessment of the effect of melatonin administration on reproduction during the use of an assisted reproductive protocol	The study group showed a higher percentage of pregnancies and live births	Melatonin may be a fertility-enhancing drug in women
Mokhtari et al. (26)	*in vivo* human female	Double-blinded randomized clinical trial	Assessment of the effect of melatonin administration on the fertility of women suffering from PCOS	The study group showed a higher percentage of pregnancies	Melatonin can potentially increase a chance of pregnancy
Celik et al. (27)	*in vivo* human female	Clinical experiment	Determining the relationship between melatonin concentration and the occurrence of cholestasis in pregnant women	Lower melatonin concentration was observed in the group of women with cholestasis	Melatonin concentration may be a predictor of the occurrence of maternal cholestasis
Hobson et al. (28)	*in vivo* human female	Clinical experiment	Assessment of melatonin use in pre-eclampsia	Blood pressure was lower in the study group	Melatonin can be used in pre-eclampsia to lower blood pressure
Zhang et al. (29)	*in vivo* animal (mouse) female	Animal medical experiment	Evaluation of the effect of melatonin concentration on female reproductive performance	Melatonin deficiency reduces the gonads and reproductive potential of females	The decreased melatonin concentration reduces the reproductive potential
Lv et al. (30)	*in vivo* animal (mouse) female	Animal medical experiment	Assessment of the effect of melatonin and leptin on female reproductive potential	Blocking the melatonin mt1 receptor causes a decrease in the concentration of leptin and steroid hormones	Melatonin is involved in leptin-mediated regulation of steroid hormone level
Lombardo et al. (31)	*in vivo* animal (Fundulus heteroclitus) female	Animal medical experiment	Evaluation of the influence of melatonin concentration on the survival of the embryo	There is a positive correlation between melatonin concentration and embryo survival	The higher the melatonin concentration, the higher the probability of embryo survival
Zhang et al. (32)	*in vivo* animal (mouse) female	Animal medical experiment	Evaluation of the influence of the MTNR1A polymorphism on the reproductive potential of females	Melatonin has a positive effect on reproduction	Higher melatonin levels may positively affect fertility
Cosso et al. (33)	*in vivo* animal (ewe lamb) female	Animal medical experiment	Evaluation of the effect of melatonin administration on female reproduction	Higher fertility and an earlier first estrus were shown in the study group	Melatonin may be a fertility enhancer for llamas
Song et al. (34)	*in vivo* animal (mice) female	Animal medical experiment	Evaluation of the effect of melatonin administration on female reproduction	The study group showed a decrease in ovarian aging, higher fertility and oocyte quality	Melatonin may increase fertility in mice
Dholpuria et al. (35)	*in vivo* animal (camel) female	Animal medical experiment	Evaluation of the effect of melatonin-releasing implants on female fertility	The study group showed a higher percentage of pregnancies	Melatonin may be a fertility drug for camels
Peng et al. (36)	*in vivo* animal (pig) female	Animal medical experiment	Assessment of the effect of melatonin on the course of pregnancy	Melatonin increases the expression of antioxidant factors	Melatonin supplementation may have a positive effect on the maintenance of pregnancy
Arend et al. (37)	*in vivo* animal (swine) female	Animal medical experiment	Evaluation of the effect of melatonin administration on female reproduction	The study group showed a higher percentage of surviving pregnancies	Melatonin may be a fertility enhancer in pigs
Bai et al. (38)	*in vivo* animal (sheep) female	Animal medical experiment	Assessment of the effect of melatonin on the immune system functioning in pregnant females	Cd4 and mtnr1a expression was higher in samples taken from the immune system in early pregnancy	Melatonin has been linked to changes in the immune system functioning during pregnancy
Gunwant et al. (39)	*in vivo* animal (water buffalo) female	Animal medical experiment	Determination of the influence of MTNR1A receptor polymorphism on reproductive potential	The reproductive potential varies depending on the MTNR1A gene variant	Melatonin receptor polymorphism affects the occurrence of fertility periods
Fathy et al. (40)	*in vivo* animal (sheep breeds) female	Animal medical experiment	Assessment of the influence of MTNR1A and AA-NAT polymorphism on reproductive potential	The reproductive potential varies with the polymorphism	The MTNR1A and AA-NAT polymorphism affects the age of the first estrus
Mura et al. (41)	*in vivo* animal (Sarda ewes) female	Animal medical experiment	Assessment of the influence of MTNR1A polymorphism on reproductive potential	The reproductive potential varies depending on the MTNR1A gene variant	MTNR1A polymorphism affects fertility
do Nascimento Marinho et al. (42)	*in vivo* animal (rats) female	Animal medical experiment	Determination of the protective effect of melatonin when exposed to pesticides during pregnancy	The genetic changes caused by exposure to cypermethrin were reduced in the study group	Melatonin can be used as a drug to protect against the negative effects of cypermethrin, but it is not effective in reversing the effect of methomyl
Huang et al. (43)	*in vivo* animal (mouse) female	Animal medical experiment	Determination of the melatonin protective effect on the ovaries during the use of cisplatin	Ovarian toxicity of cisplatin is decreased when melatonin is used during chemotherapy	Melatonin can be used in a protective way during cisplatin administration to preserve fertility
de Almeida (44)	*in vivo* animal (rats) female	Animal medical experiment	Evaluation of the effect of melatonin on fertility after exposure to herbicides in female rats	The study group showed a higher percentage of pregnancies and fetal survival	Melatonin can be used as protection against herbicide exposure
de Sousa Coelho et al. (45)	*in vivo* animal (rats) female	Animal medical experiment	Determination of the protective effect of melatonin on the fetus exposed to ethanol	Melatonin reduces the defects caused by exposure of the fetus to ethanol	Melatonin can be used to prevent fas
Wang et al. (46)	*in vivo* animal (mouse) male	Animal medical experiment	Determination of the melatonin protective effect on the testes during the use of paclitaxel	The sperm quality increases after the use of melatonin during treatment with paclitaxel	Melatonin can be used in a protective way during paclitaxel administration to preserve fertility
El Gheit et al. (47)	*in vivo* animal (rat) male	Animal medical experiment	Evaluation of the effectiveness of melatonin in the treatment of varicocele	Less histopathological changes in the testicle were found in the melatonin group	Melatonin can be used as an adjunct therapy for varicocele to reduce damage to the testes

Other hormones produced by the anterior pituitary gland include adrenocorticotropic hormone (ACTH) and growth hormone (GH). ACTH is produced from the precursor hormone proopiomelanocortin (POMC), which stimulates the production of cortisol in the adrenal glands *via* melanocortin receptors. Corticotropin-releasing hormone (CRH), along with vasopressin (ADH), are the main hormones that control ACTH secretion. GH and ACTH secretion is pulsatile and subject to circadian rhythms. Factors that influence the increase in growth hormone secretion are sleep and physical activity, as well as fasting, hypoglycemia, hypovolemia, and surgery. Hyperglycemia initially reduces and then increases GH levels (rebound effect). GH secretion shows gender differences, in males it is pulsatile and in females secretion is continuous. GH levels decline with age and somatopause occurs in the elderly (24).

### 3.1 Female

#### 3.1.1 *In vivo* human studies

Growth hormone, by activating insulin-like growth factor 1 (IGF-1) synthesis, directly improves the quality of oocytes, and also increases FSH-induced ovarian steroidogenesis. Administration of GH during ovulation stimulation enhances the effectiveness of the in-vitro procedure by increasing the percentage of mature oocytes and embryos viable to the day of transfer. Daily administration of a low dose of growth hormone (0.5 IU) started on the day of GnRH agonist administration increases the pregnancy rate (34.4% vs. 0%), the percentage of good-quality embryos and cryopreserved embryos. In addition, the use of a low dose of GH (4IU/d) in patients with reduced response to GnRH antagonists preparing for the IVF/ICSI procedure decreases the effective dose of gonadotropins and the duration of stimulation, increasing the total number of oocytes and oocytes in metaphase II of meiosis (48).

In obese women, reduced fertility results, among others, from decreased LH levels. They experience accelerated sexual maturation, menstrual disorders, in particular prolongation of the follicular phase, indicating ovulation disorders, and a higher frequency of obstetric complications, including spontaneous miscarriages (49).

Interestingly, the serine protease inhibitor (SERPINA12, also known as Vaspin) plays a significant role in the pathogenesis of type 2 diabetes, inflammation and infertility. Its concentration increases with weight gain and the worsening of insulin resistance, and decreases with weight loss. Metformin reduces the serum concentration of Vaspin in patients with PCOS (50).

Too low body weight, intense exercise, chronic diseases, malabsorption disorders such as celiac disease, hyperthyroidism, and thus energy deficiencies lead to impaired pulsatile GnRH secretion, and as a result, the concentration of LH and FSH necessary for effective stimulation of the gonads is too low (51). Studies have also shown that women with a BMI below 18.5 have a 72% higher risk of miscarriage in the first trimester of pregnancy than women with a normal body weight (52).

Disturbance of the circadian rhythm including sleep quality, wakefulness and the above-related pulsatile hormone secretion plays a significant role in infertility. It is known that insomnia in female shift workers results in decreased melatonin production. Low melatonin concentration in Graaf vesicles is associated with higher generation of free oxygen radicals (ROS), and thus poorer quality of oocytes. Melatonin supplementation in patients undergoing the *in vitro* procedure increases its effectiveness by improving the quality of oocytes, increasing the percentage of fertilization and the quality of the emerging embryos (24).

Prolactin is a peptide hormone synthesized by lactotrophic cells of the anterior pituitary gland. Its secretion is mainly influenced by dopamine, which negatively influences its secretion through negative feedback. The main function of prolactin is to influence the development of the mammary glands during pregnancy and to support milk production after delivery. Plasma prolactin levels increase rapidly during pregnancy with an increase in the size and number of the anterior pituitary lactotrophic cells. During breastfeeding, the sucking of the nipple by the baby causes a rapid secretion of prolactin through a mechanism of rapid neuroendocrine reflex. In pathological situations, the symptoms of hyperprolactinemia include hypogonadotropic hypogonadism (menstrual disorders and infertility) or visual field disturbances due to the mass effect caused by prolactinoma, the most common pituitary tumor. Hyperprolactinemia is diagnosed by measuring plasma prolactin. A false-positive result may be due to the presence of macroprolactin in the plasma. Its verification is possible with the use of the polyethylene glycol (PEG) precipitation method, which allows to avoid unnecessary treatment (53).

Hypothyroidism leads to a compensatory increase in the production of thyrotropin-releasing hormone (TRH) by the hypothalamus, which in turn results in suppression of dopamine production, resulting in excessive prolactin secretion, i.e. hyperprolactinemia (54). Central nervous system (CNS) tumors that produce prolactin, called prolactinomas, also cause hyperprolactinemia. Hyperprolactinemia is of key importance in infertility as it inhibits the production of GnRH and, consequently, also of gonadotropins (LH, FSH) by the pituitary gland. The therapy most often uses bromocriptine, while the basic diagnostic imaging test is magnetic resonance imaging (MR) in the T1 sequence (19, 20, 55). Prolactinomas are the most common hormonally active tumors in pregnant women. A case of a 30-year-old woman in the 36th week of pregnancy with headaches and left-sided vision loss was described. MRI of the pituitary gland confirmed a 10x11mm suprasellar lesion. After delivery, the lesion was removed by endoscopic transnasal resection. Histopathological examination revealed a tissue of prolactinoma. Therefore, a number of potential causes, including prolactinoma, should be considered in the differential diagnosis of visual loss during pregnancy (56).

Studies on the neuropeptide kisspeptin encoded by the Kiss1 gene prove that it is a potent inducer of GnRH production. In animal models, mice with hyperprolactinemia were administered kisspeptin once a day for 20 days, which led to the restoration of estrus cycles and an increase in FSH and LH levels. Thus, kisspeptin may be used in the future to treat infertility associated with hyperprolactinemia (54). In studies with mice and sheep, paracrine substances secreted by the uterine glands have been shown to play a key role in implantation of the embryo and maintenance of pregnancy in the early stages. Therefore, problems with getting pregnant and with its maintenance may result from impaired functioning of the uterine glands. A thorough understanding and study of this issue may in the future contribute to improving the outcomes of infertility treatment as well as the maintenance of health by the mother and the fetus (57).

Multi-hormonal pituitary insufficiency is most often idiopathic and is associated with a mutation in one or several genes, among which the most important are transcription factors such as GLI2, LHX3, LHX4, HESX1, PROP1, POU1F1, SOX2, PITX2, OTX2, SOX3. Clinically, it is possible to encounter an isolated deficiency of one of the pituitary gland hormones or with a disturbance in the production of some or even all pituitary hormones, including gonadotrophins. It is therefore another potential cause of infertility (58). Postpartum pituitary hemorrhage, known as Sheehan’s syndrome, is a common cause of multi-hormonal pituitary insufficiency in tropical countries. Hypopituitarism also occurs in 10% of survivors of a poisonous snake. In turn, post-traumatic pituitary insufficiency accounts for approximately 7% of all cases (59). Empty sella syndrome can be another cause of hypopituitarism. It may be idiopathic or associated with postpartum hemorrhage, head trauma, CNS stroke, hormonally active pituitary microadenoma, and after radiation therapy or surgery. It manifests itself clinically with headaches, visual disturbances and hormonal insufficiency of the pituitary gland (60).

Autoimmune diseases, especially systemic lupus erythematosus (SLE) and rheumatoid arthritis (RA), as well as the toxicity of disease-modifying drugs, in particular non-steroidal anti-inflammatory drugs (NSAIDs) and glucocorticoids (GCs), also affect infertility. Although recently there has been an improvement, especially thanks to the use of biological drugs that reduce disease activity to a greater extent (61).

Infections in the hypothalamic-pituitary region account for less than 1% of the causes of its damage. The etiological factors can be bacteria, viruses, fungi and parasites. In imaging studies, infection may appear as a tumor in the area of ​​the sella turcica, which raises the suspicion of a proliferative process. Risk factors for infection include meningitis, sinusitis, neurosurgery, and immune system disorders. Infection may develop in glands unchanged or damaged by previous diseases (adenomas, Rathke’s cyst, craniopharyngioma). Diagnostics is difficult due to the lack of specific symptoms. Patients may experience blurred vision or headache, fever and leukocytosis. During the acute phase of the disease, but also several months or years after successful antibiotic therapy, a significant proportion of patients develop symptoms of hypothalamic-pituitary insufficiency (62). Hypopituitarism can be caused by inflammation of the pituitary gland and can be primary or secondary. The most common form of primary inflammation is lymphocytic pituitary inflammation, which is most frequent in pregnant or postpartum women. In turn, immunotherapy, used in the treatment of neoplastic diseases, is important in secondary pituitary inflammation. Autoimmune complications, which can also affect the pituitary gland, are a side effect of such treatment. The most common clinical symptoms are headache, excessive thirst and visual disturbances, while laboratory deficiencies include ACTH, TSH, FSH, LH, GH and hyperprolactinemia. Although biopsy remains the gold standard in the diagnosis of pituitary inflammation, the most important in clinical practice is MRI, which also helps to differentiate pituitary inflammation from pituitary adenomas (63).

#### 3.1.2 *In vivo* animal studies

It is known that stress negatively influences the homeostasis of the circadian rhythm, which results in disturbance of the hypothalamic-pituitary-gonadal axis. In animal models, knock-out of clock genes such as period circadian regulator 1 and 2 (Per1, Per2), brain and muscle Arnt-like protein-1 (Bmal1) led to infertility in mice (64).

The female reproductive system is also affected by toxic substances such as pesticides, heavy metals, diethylstilbestrol, phenols, bisphenols, parabens. They can interfere with receptor binding, steroidogenesis and hormone metabolism. They also have a proven effect on the increased incidence of preterm labor, fetal growth disorders, miscarriages and difficulties in getting pregnant. Animal studies have shown that exposure to pesticides caused damage to oocytes, decreased production of steroid hormones by the ovaries, and lower fertility. Exposure to diethylstilbestrol suppressed gonadotropin production and caused damage to the pituitary gland and uterine tissue (65).

#### 3.1.3 *In vitro* human studies

*In vitro* studies have shown that cannabinoids influence the functioning of the hypothalamus and pituitary gland through CB-1 receptors. Blocking these receptors results in an increase in the concentration of ACTH, GH, LH, and TSH, which proves that cannabinoids have an inhibitory effect on the activity of the hypothalamic-pituitary axis (66).

### 3.2 Male

#### 3.2.1 *In vivo* human studies

Gonadotropin-releasing hormone produced by the hypothalamus regulates the release and secretion of gonadotrophins that control the testes functioning, i.e. luteinizing hormone and follicle-stimulating hormone from the anterior pituitary. FSH receptors are located on Sertoli cells and LH receptors on Leydig cells. The effect of gonadotropins on testicular cells determines the proper course of testosterone synthesis, spermatogenesis, and the quality of sperm. The functioning of the male reproductive system is also influenced by hormones such as estradiol (E2) and prolactin. Estradiol, produced both by the testes and by the peripheral conversion of testosterone, is a potent inhibitor of LH and FSH. On the other hand, prolactin, by inhibiting the production of GnRH, reduces the concentration of LH and testosterone, which results in hypogonadism. In response to the generation of free oxygen radicals (ROS) by the male reproductive system cells, the hypothalamic-pituitary-adrenal axis is activated and cortisol is produced in response to stress. Cortisol, in turn, inhibits the production of gonadotropins, which causes a decrease in the testosterone production by Leydig cells (due to the lowered LH levels). Moreover, low FSH levels reduce the release of androgen-binding protein (ABP) by Sertoli cells, which further decreases circulating testosterone levels. Interestingly, obesity affects not only the hypothalamic-pituitary-adrenal and hypothalamic-pituitary-thyroid axes, but by generating ROS, it affects the increased production of leptin by adipocytes, which, together with insulin, reduces the concentration of triiodothyronine (T3), and thus adversely affects the testes functioning. Leptin is also an inhibitor of GnRH production by the hypothalamus (67). The pre-pubertal rise in leptin levels is responsible for the proper testes development. In turn, an increase in androgen concentration in adolescent boys causes a decrease in leptin concentration (68).

Obesity is not only associated with a higher risk of cardiovascular disease, stroke, type 2 diabetes, but also with disorders in the reproductive system in both women and men. It also leads to hyperinsulinemia, hyperlipidemia, hyperleptinemia and chronic inflammation. In obese men, decreased levels of testosterone, luteinizing hormone and sex hormone-binding globulin (SHBG) are observed. Increased amount of adipocytes negatively affects the quality and quantity of sperm, and as BMI increases, total sperm count decreases. A meta-analysis of 21 studies involving 13,000 men found a higher incidence of oligozoospermia and azoospermia in obese men. Moreover, the effectiveness of the IVF procedure decreases when the partner is obese (21).

In addition, studies in rats have shown that sleep disorders reduce testosterone levels, decrease sperm motility and apoptosis of Leydig cells in the testes. Moreover, it had a negative effect on the initiation of sexual behavior in males and on the percentage of ejaculation (69).

Infection with the severe acute respiratory syndrome coronavirus 2 (SARS-CoV2) is the cause of a cytokine storm that damages many organs. In addition to the well-known effects of SARS-CoV2 infection on the lungs, heart, and CNS, testicular damage is also under discussion. Evidence for the presence of SARS-CoV2 in sperm samples and in testicular tissue is limited, while an impaired immune system response, hyperthermia and oxidative stress caused by infection, damage testicular tissue. Testicular steroidogenesis defects related to SARS-CoV2 infection are the cause of decreased testosterone concentration, which leads to impaired spermatogenesis, impotence and, consequently, infertility in a significant percentage of convalescents. Increased levels of FSH and LH are biomarkers of testicular damage. Hypothalamic-pituitary-gonadal dysregulation due to SARS-CoV2 infection is also associated with the risk of acute renal failure and cirrhosis (70). It should also be noted that HIV infection is associated with an increased risk of pituitary gland stroke as well as pituitary lymphoma and may therefore lead to hypopituitarism, one of the effects of which is infertility (71).

## 4 Pineal gland

The pineal gland is a relatively small gland located within the cranial cavity behind the third ventricle that connects to the cerebrum by a peduncle. It is a highly vascularized part of the brain not covered by the blood-brain barrier. 90-95% of the cells of this gland are pinacocytes, whose main function is the production of melatonin (N-acetyl-5-methoxytryptamine) (72). Its synthesis is regulated by the superior cervical ganglion, which receives information about the intensity of light through the suprachiasmatic nuclei of the hypothalamus, considered to be the anatomical center of the biological clock (73). The pineal gland, through melatonin, is responsible for controlling the regulation of the circadian rhythm, mainly sleep and wakefulness. Its synthesis and secretion significantly increases in the dark (74), and exposure to a light source causes a significant reduction in its synthesis (75). Melatonin acts through the MT1 and MT2 receptors located in the suprachiasmatic nucleus (SCN), reducing the activity of its neurons, which ultimately leads to the feeling of drowsiness and falling asleep (76). Another important function of melatonin is the inhibition of sexual maturation. In humans, the decline in the average daily melatonin production progresses with age and is associated with the progression on the Tanner scale (77, 78). This is due to the inhibition of kisspeptin expression by melatonin, which stimulates the hypothalamus cells to synthesize GnRH (79, 80). It has been proven that the administration of exogenous melatonin causes delayed puberty in children of both sexes (81). Melatonin is also produced in other organs, including the reproductive system (82–87), the retina and lens (88), the gastrointestinal tract (89, 90) and blood cells (91), acting as an autocrine or paracrine hormone (90). However, it most likely does not significantly affect the plasma concentration of melatonin as it was found to be undetectable in rats after pituitary removal (92). Melatonin is also considered to be a very effective antioxidant (73). By activating its MT1 and MT2 receptors, it stimulates the expression of superoxide dismutase (SOD), catalase (CAT), glutathione peroxidase (GPx), and glutathione reductase (GRd) (93). Another mechanism of antioxidant activity is the stimulation of the Nrf2 expression, which is a transcription factor for many genes encoding antioxidant enzymes (94). There are also many reports on the direct antioxidant activity of its metabolites: N1-acetyl-N2-formyl-5-methoxykynuramine (AFMK), N1-acetyl-5-methoxykynuramine (AMK) and cyclic 3-hydroxymelatonin (3-OHM) (95).

### 4.1 Female

#### 4.1.1 *In vivo* human studies

Melatonin appears to be an important hormone related to female fertility. Its higher plasma levels have been found in women with infertility caused by hypothalamus dysfunction (96, 97). At the same time, it has been shown that melatonin supplementation can positively affect fertility and improve the effectiveness of assisted reproductive protocols (25), which may be related to its influence on the increase of melatonin concentration in the follicular fluid (25, 98). Due to its antioxidant effect, melatonin has a positive effect on the quality of oocytes. Negative effects on fertility may be related to the inhibition of GnRH expression, which promotes ovulation by stimulating LH expression. Melatonin supplementation has also been shown to be beneficial in women suffering from polycystic ovary syndrome (26). This is due to a decrease in plasma testosterone concentration and elevated probability of ovulation (99). Studies carried out in a group of transgender men showed that testosterone inhibited the expression of gonadotropic hormones, which contributed to a strong suppression of ovulation (100). The reasons for the positive effect on the fertility of melatonin supplementation in PCOS patients are likely to be found in the antioxidant potential decrease (101).

Melatonin is associated not only with fertility, but also with the course of pregnancy. An increase in melatonin concentration during pregnancy and its sudden decrease after delivery have been shown (102). Furthermore, an increased risk of cholestasis has been demonstrated in women with a lower melatonin concentration (27) and the effectiveness of melatonin administration in lowering blood pressure and extending the duration of pregnancy in the course of pre-eclampsia has been confirmed (28). Studies are still needed to establish the relationship between melatonin levels and the course of pregnancy in more detail.

#### 4.1.2 *In vivo* animal studies

In animals, a strong relationship between melatonin and fertility has also been demonstrated. Melatonin deficiency is correlated with decreased fertility (29, 30), lower levels of sex steroid hormones (30), and decreased survival of embryos after fertilization (31). At the same time, a positive correlation was observed between serum melatonin concentration and fertility (32–36). Administration of exogenous melatonin is associated with a higher percentage of maintained pregnancies (37). Melatonin also participates in the activity changes of the female immune system during pregnancy in sheep, influencing the immune activity of the thymus, lymph nodes and spleen, which are involved in the process of fetal immune tolerance. The effect of melatonin on the immune system is complex and pleiotropic. At the beginning of the inflammatory reaction, it is immunostimulating, later it changes to immunosuppressive. The changes induced by melatonin in pregnancy are mainly related to the stimulation of the helper lymphocyte population by directly affecting the MT1 and MT2 receptors (38).

The relationship between melatonin receptors polymorphism and female fertility has been proven (32, 39–41). There are also numerous reports suggesting a protective effect of melatonin administration on fertility, maintenance of pregnancy and prevention of birth defects in females exposed to cytostatics, insecticides, herbicides and alcohol (42–46), which is directly related to its antioxidant nature.

#### 4.1.3 *In vitro* human studies

*In vitro* studies focus mainly on the antioxidant activity of melatonin and its relationship with the production of gonads. In females, a positive correlation was demonstrated between the melatonin concentration in the follicular fluid and the markers of the ovarian reserve and the quality of oocytes (103–105).

#### 4.1.4 *In vivo* animal studies

Similarly in animal studies, the melatonin level in the follicular fluid correlated with the quality of oocytes and ovarian reserve (106, 107). In addition, melatonin seems to be a good component of cryopreserves used in the storage of the oocytes (108–110). The induction of estradiol production by granular cells was also confirmed (106).

### 4.2 Male

#### 4.2.1 *In vivo* human studies

In men, melatonin is also very important in terms of fertility. Similarly to women, melatonin causes a decrease in LH secretion, leading to lower testosterone secretion by Leydig cells in the testes, which has a negative impact on the intensity of spermatogenesis (107). A positive correlation was demonstrated between the concentration of melatonin and male fertility, both in the serum and in semen. Interestingly, an increased concentration of melatonin in the plasma and its decreased level in semen was demonstrated in infertile men (111). Studies have shown that the concentration of melatonin in semen inversely correlates with the frequency of sperm DNA damage, which is probably due to its antioxidant activity (112). It has also been reported that melatonin supplementation improves sperm quality (113, 114) and increases the chance of preserving fertility in patients with varicocele (115).

#### 4.2.2 *In vivo* animal studies

There are also studies linking melatonin to male fertility in animals (46). Protective effect on gonads has been demonstrated in the course of varicocele, radiotherapy and metabolic syndrome, exposure to cytostatics and heavy metals (47, 116–119). In addition, the synthesis of melatonin within the testes has been confirmed (120) and the positive effect of its concentration on semen quality was demonstrated (121).

#### 4.2.3 *In vitro* animal studies

The importance of the antioxidant activity of melatonin during spermatogenesis has also been shown in animal studies (122). Furthermore, melatonin seems to be a good component of cryopreserves used in the storage of sperm samples (123).

## 5 Thyroid

### 5.1 Female

#### 5.1.1 *In vivo* human studies

Inhibition of the secretion of thyroid hormones is associated with its hypothyroidism. In milder hypothyroidism, infertility is usually not achieved, but the risk of spontaneous miscarriage, premature births and stillbirths increases (124–126) (**Table 3**). Severe hypothyroidism may lead to infertility as a consequence of a direct inhibitory effect on the ovarian ovulatory activity as well as through an effect on the pituitary-ovarian axis. Decreased activity of sex hormone-binding globulin causes an increase in serum free testosterone and estradiol, moreover, metabolic clearance of androstenedione and estrone is decreased. The elevated level of thyrotropin-releasing hormone in primary hypothyroidism is responsible for the increase in serum prolactin levels and the delayed luteinizing hormone response to the stimulating effect of gonadotropin-releasing hormone, which in turn leads to luteal phase failure in women (124–126, 131, 132).

**Table 3:** Thyroid-related fertility studies.

Authors (reference)	Type of study	Study design	Aim	Results	Conclusion
Vaquero et al. (126)	*in vivo* human female	Clinical trial	Evaluation of the role of benign thyroid abnormalities in recurrent spontaneous abortion and evaluation of the effects of two different therapeutic protocols	Treatment of mild thyroid disorders with immunoglobulins resulted in termination of pregnancy in 54.5% of cases, while the use of replacement therapy in 81.2% of cases	Thyroid replacement therapy is more effective in the obstetric context than intravenous immunoglobulin
Kakita-Kobayashi et al. (127)	*in vitro* human female	Clinical experiment	Evaluation of the effect of thyroid hormone on decidualization in human endometrial stromal cells (hescs) and determination of its physiological roles *in vitro* through gene targeting	A significant increase in decidual response was observed after combined treatment with ovarian steroid hormones and thyroid hormone	Deciduality impairment is a possible cause of infertility in patients with subclinical hypothyroidism (SCH)
Barber et al. (128)	*in vitro* human female	Clinical experiment	Determination of the expression of thyroid hormone receptors in the extranodal trophoblast, elucidation of the effect of T3 on both the proliferation and differentiation of human trophoblast cells of various origins, and the determination of a potential interaction between EGF and T3	T3 and EGF have an anti-proliferative effect on cells of the extranodal-like lineage (SGHPL-4), with a concomitant proliferation-promoting effect on JEG-3 choriocarcinoma cells	EGF and T3 have a synergistic effect in regulating human trophoblast proliferation and differentiation.
Giuliani et al. (129)	*in vitro* animal (rat) female	Clinical experiment	Evaluation of the effect of quercetin on the expression of genes encoding thyroid hormones and radioiodine uptake	Quercetin reduces the expression of the thyrotropin receptor, thyroid peroxidase and thyroglobulin genes, and also reduces the uptake of radioiodine by thyroid cells	Quercetin may act as a thyroid disruptor, supplementation should be used with caution
Condorelli et al. (130)	*in vitro* human male	Clinical experiment	Assessment the *in vitro* effects of levothyroxine (LT4) on conventional and biofunctional sperm parameters and its implications on fertility	Thyroid hormones have a beneficial effect on sperm mitochondrial function, oxidative stress and DNA integrity	Restoring the normal secretion of thyroid hormones is important in idiopathic male infertility

#### 5.1.2 *In vitro* human studies

Due to the small number of *in vitro* studies and their contradiction regarding the influence of the role of thyroid hormones on reproductive function and semen parameters in humans, it is necessary to exercise caution when interpreting them. Kakita-Kobayashi et al. determined the effect of levothyroxine on decidualization in human endometrial stromal cells (hESCs) and the selection of key genes for this process *in vitro*. After exposure of cells to the drug, a significant increase in the temporal response was observed. Moreover, treatment with LT4 also influenced the regulation of many transcription factors important for decidualization. There was also an increase in type 3 deiodinase in the presence of thyroid hormones, which is an important element in the tissues of the fetus and placenta. In addition, it has been noted that the progesterone receptor and the ovarian steroid hormone receptor are involved in thyroid hormone-induced decidualization. Based on the conducted study, Kakita-Kobayashi et al. claim that impaired decidualization is a possible cause of infertility in patients with subliminal hypothyroidism (127).

It was shown that the trophoblast contains triiodothyronine receptors. *In vitro* studies indicate that thyroid hormones have a direct impact on the early development of the placenta by stimulating angiogenesis and by promoting the invasion and differentiation of embryonic cells (133). Thyroid receptors are found in the endometrium, and their highest levels are observed in the receptive endometrium (134). Barber et al., using immunohistochemical staining, localized specific isoforms of thyroid hormone receptors in extracellular trophoblasts in placenta biopsies in the first and second trimesters of pregnancy, which indicates a potential sensitivity of cells to T3. Epidermal growth factor (EGF) and T3 exhibited antiproliferative effects on the extravillous trophoblast cell line (SGHPL-4) while promoting proliferation in JEG-3 chorionic cancer cells. The study suggests that T3 and EGF may act synergistically, regulating both the proliferation and the differential function of human trophoblast (128).

#### 5.1.3 *In vitro* animal studies

In 2008, quercetin was shown to inhibit the dose-and time-dependent spread of Fischer rat thyroid cell line (FRTL-5) by inhibiting the insulin-regulated action of Akt kinase. Quercetin interferes with TSH-dependent NIS gene expression and transport in FRTL-5 cells. These observations may help to understand the molecular mechanism of quercetin’s anti-thyroid effects on cell growth and function. Even when taken from an *in vitro* thyroid cell line that does not have the characteristics of a transformed cell, these results led to the evaluation of quercetin as an anti-thyroid drug in hyperthyroidism (135, 136). In recent studies, quercetin appears to reduce the expression of the thyroid-stimulating hormone receptor, thyroid peroxidase (TPO) and thyroglobulin (Tg) genes. The anti-thyroid effect of quercetin was further assessed *in vivo*. It was administered (50 mg/kg) to a Sprague-Dawley rat, and after 14 days of treatment, the radioiodine uptake decreased significantly, indicating that quercetin may act as a thyroid disruptor (129, 135). Soybean extracts inhibit iodine uptake and increase the protein content of a known autoimmune Tg fragment in rat Fischer thyroid cells. These effects may be responsible for the link between more frequent soy consumption and thyroid disorders such as hypothyroidism, goiter, and autoimmune thyroid disease (135, 137). Among the flavonoids, epigallocatechin-3 gallate (EGCG), a catechin rich in green tea, when administered to male rats at doses of 25, 50 and 100 mg/kg of body weight, showed an antithyroid activity, which was manifested by reduced activity of thyroid peroxidase and 5′-deiodinase I and increased activity of the thyroid sodium-potassium pump. In addition, serum T3 and T4 levels were lowered while serum TSH was elevated in rats, demonstrating the possibility of goiter *in vivo* (135, 138).

### 5.2 Male

#### 5.2.1 *In vitro* human studies

Condorelli et al. assessed the effects of levothyroxine (LT4) on conventional and biofunctional sperm parameters and their impact on fertility. The authors obtained sperm from men with confirmed infertility and exposed them to LT4 *in vitro*. The results confirmed the effect of LT4 on an increase in the number of sperm with a high level of mitochondrial membrane potential (MMP), a reduction in the percentage of sperm with a low MMP and an increase in sperm motility. L4 was found to induce sperm proliferation and lipid peroxidation, thereby improving the chromatin tightness in the cell nucleus. According to the researchers, the results of the above-mentioned *in vitro* study may have clinical application in patients with idiopathic infertility due to the explanation of the influence of thyroid function on fertility in men (130).

So far, a key role of vitamin D in the male reproductive system has been suggested as its receptors and metabolizing enzymes have been shown to be expressed in the testes and sperm. Vitamin D metabolism is mainly regulated by parathyroid hormone (PTH), produced by the parathyroid glands, and fibroblast growth factor 23 (FGF23), synthesized by osteoblasts and osteoclasts. Decreased levels of circulating calcium and 25-hydroxyvitamin D3 increase PTH secretion, which stimulates 1-α-hydroxylase and inhibits the expression of 24-hydroxylase in the kidney, leading to higher levels of vitamin D and calcium (139, 140). In addition, elevated levels of phosphorus and 25-hydroxyvitamin D3 inhibit 1-α-hydroxylase and stimulate 24-hydroxylase, resulting in a reduction in vitamin D. To close the feedback loop when vitamin D and phosphorus levels decline, FGF23 is inhibited, leading to an increase in vitamin D levels (139, 141). The vitamin D receptor (VDR) and the enzymes that metabolize vitamin D are simultaneously expressed in Sertoli cells, germ cells, Leydig cells, sperm, and cells in the epithelial lining of the male reproductive system. The presence of vitamin D metabolizing enzymes suggests that the reproductive organs may modulate the local response to vitamin D in animals and humans. Nuclear somatic or embryonic cells appear to be able to synthesize and degrade vitamin D locally, independent of systemic vitamin D metabolism. Moreover, expression of VDR in the testes suggests that vitamin D may exert autocrine and paracrine effects, possibly playing a role in regulating testicular function, thus contributing to male infertility. The expression of VDR and vitamin D metabolizing enzymes in the male reproductive system has been extensively analyzed in animal and human studies. The VDR protein has been found in the prostate, seminal vesicles, epididymis, and also in germ cells, especially spermatogonia, spermatocytes, and Sertoli cells (139, 142). VDR protein expression has been found in animal sperm but has been suppressed in the tail of the epididymis (139, 143). In the same context, testosterone synthesis enzymes in the testes appeared to be reduced in mice fed the vitamin D deficiency (VDD) diet (144).

## 6 Thymus

Changes in the immune system activity are necessary in the course of a healthy pregnancy as they help to avoid an immune response against an allogeneic fetus (145) (**Table 4**). One of the organs whose activity change is required for the development of immune tolerance during pregnancy is the thymus, in which the maturation and differentiation of helper T cells take place.

**Table 4:** Thymus-related fertility studies.

Authors (reference)	Type of study	Study design	Aim	Results	Conclusion
Saito et al. (146)	*in vivo* human female	Comparative study	Assessment of activation antigens on T cells of human decidua at an early stage of pregnancy	The decidua contained a small number of T cells and both CD4+ and CD8+ subsets expressed CD69, HLA-DR, IL-2R alpha and IL-2R beta antigens	T cells in the decidua in the first trimester of pregnancy are regionally activated
Watanabe et al. (147)	*in vivo* human female	Comparative study	Assessment of changes in T, B, and NK lymphocyte subsets during and after pregnancy	Suppressor T and NK+3 cells increase in early pregnancy and decrease in late pregnancy	The number of T, B, and NK lymphocyte subsets changes throughout pregnancy
Li et al. (148)	*in vivo* animal (sheep) female	Animal medical experiment	Assessment of TLR expression change in early pregnancy in females	Changes in TLR expression have been demonstrated in early pregnancy	Alteration of TLR expression may be involved in the generation of immune tolerance
Zhang et al. (149)	*in vivo* animal (sheep) female	Animal medical experiment (sheep)	Assessment of the influence of early pregnancy hormones on thymic cytokine expression in females	Increased expression of cytokines stimulating the production of th1 and th2 lymphocytes	In early pregnancy, the production of helper t cells that may be involved in the production of immune tolerance is stimulated
Yang et al. (150)	*in vivo* animal (sheep) female	Animal medical experiment	Assessment of the influence of early pregnancy hormones on prostaglandin synthesis in the female thymus	A large increase in thymic prostaglandin synthesis has been demonstrated	In early pregnancy, the expression of prostaglandin synthases in the thymus changes, which may be associated with the development of immune tolerance
Zhang et al. (151)	*in vivo* animal (sheep) female	Animal medical experiment	Assessment of the interferon stimulated change in protein expression during early pregnancy in females	Changes in the expression of interferon-stimulated genes have been demonstrated in early pregnancy	The altered expression of certain proteins may be associated with the development of immune tolerance during pregnancy
Wise (152)	*in vivo* animal (rat) male	Animal medical experiment	Assessment of the effects of thymulin on rat testicular steroid synthesis	Increased doses of thymulin (100 ng/ml) resulted in decreased testicular steroid synthesis	Increased concentrations of thymulin had inhibitory effects on testicular steroidogenesis
Wise and Ford (153)	*in vivo* animal male	Animal medical experiment (boar)	Assessment of the effects of thymic peptide thymulin on testicular steroid synthesis	Injection of thymulin increased circulating testosterone concentrations	Thymulin increases androgen stimulation in boar testes
Jacobo et al. (154)	*in vivo* animal (rat) male	Animal medical experiment	Analysis the phenotype and number of T lymphocytes in the testicular interstitium of rats during EAO development	CD4+Foxp3+ T(reg) cells were more abundant than CD8+Foxp3+ T(reg) cells	The numbers of T(reg) cell subsets increased in the testis of rats with orchitis

### 6.1 Female

#### 6.1.1 *In vivo* human studies

Maternal Th, Tc and NK cells express activation markers on their surface after blastocyst implantation in the uterine endometrium, which suggests rapid recognition of trophoblast by these cell populations (146). Many studies have shown changes in the population of T lymphocytes and their activity in normal pregnancy (147, 155–160). In addition, human placenta produces IL-35 with an immunosuppressive effect, stimulating the proliferation of regulatory T cells in the thymus (161, 162).

The activity of the thymus is also important in terms of the etiology of primary ovarian insufficiency (POI), where one of the causes may be an autoimmune reaction. An increased risk of POI in patients with autoimmune diseases and an increased risk of autoimmune diseases in patients with POI have been observed (163).

#### 6.1.2 *In vivo* animal studies

In animal studies, changes in the population of T lymphocytes mainly include an increase in the Th cell population during early pregnancy (149), a greater percentage of type 2 helper cells in relation to type 1 helper cells (148, 164), changes in the toll-like receptor (TLR) expression within thymic epithelial cells, and the synthesis of prostaglandins and proteins stimulated by interferon (150, 151). These changes are directly responsible for the phenomenon of fetal immunotolerance, inhibiting the development and activation of a subpopulation of T lymphocytes that can stimulate inflammatory reactions within the uterine endometrium, while maintaining the endometrial and trophoblast defense against pathogens (165). This is possible due to the activity of the placenta, which produces Th2-specific cytokines such as IL-4, IL-5 and IL-10 throughout the duration of pregnancy (164).

During pregnancy, there is both local (within the uterus) and general increase in the population of regulatory T cells specific for paternal antigens (166, 167), which is responsible for the inhibition of the immune response to them.

The activity of thymus is related not only to pregnancy, but also to the maturation of the ovaries and their activity. Studies in mice have shown that early removal of the thymus causes disorders in the ovarian development, failure of maturation and sterility. This effect was reversible by administering thymulin, although a decrease in fertility was observed due to the presence of autoreactive T cells (168, 169). It is related to the influence of thymulin on the expression of LH and FSH by the pituitary gland. It has been demonstrated that GnRH and thymulin act synergistically with the release of LH and additively with the FSH secretion (170). The variable effect of thymulin on LH and FSH expression depending on the time of the menstrual cycle has also been reported (171).

### 6.2 Male

#### 6.2.1 *In vivo* animal studies

A positive correlation was demonstrated between the concentration of thymulin and LH, as well as a reduction in testosterone production with a decrease in thymulin concentration (152, 153). The presence of regulatory T cells within the testes has also been demonstrated (154, 172), which, through the secretion of IL-10, affect the ratio of the Th1 to Th2 subpopulation. Their presence is essential for the immunotolerance of spermatocyte antigens. In the case of disorders leading to a reduction in their population size, autoimmune orchitis may occur (154).

## 7 Pancreas

Disorders of lipid and glucose metabolism, recently associated with the increasingly frequent obesity, are a current medical problem. They not only pose a health risk, but also have an adverse effect on reproductive function (**Table 5**). Insulin resistance, i.e. reduced cell sensitivity to insulin and compensatory hyperinsulinemia, induce an early response to luteinizing hormones and cause premature differentiation of small follicles, resulting in anovulation. On the other hand, they adversely affects the functions and environment of the endometrium and are responsible for disturbances in embryo implantation (180). In addition, hyperinsulinemia disturbs the intra-follicular microenvironment during folliculogenesis, decreases the fertilization rate and the potential for embryonic development during natural ovarian stimulation cycles (173). Insulin primarily acts on its own receptors located on the theca cells surrounding the stromal and granulosa cells, thereby stimulating ovarian steroidogenesis (174). Research shows that inflammation (elevated levels of IL-6 and IL-17) accompanying insulin resistance and obesity affect ovulation and fertilization and increase the risk of early miscarriage. Moreover, obese people show increased levels of estrogens due to the overexpression of aromatase in adipose tissue, which in turn disturbs ovulation (173, 175).

**Table 5:** Pancreatic-related fertility studies.

Authors (reference)	Type of study	Study design	Aim	Results	Conclusion
Wang et al. (173)	*in vivo* human female	Prospective cohort study	Evaluation of the impact of insulin resistance on IVF outcomes in women without PCOS	The percentage of mature oocytes and the rate of blastocyst formation were significantly lower in the IR group compared to the group without IR	In lean infertile women without PCOS, insulin resistance is associated with a reduced percentage of mature eggs and poor embryo quality, in which pancreatic B-cell immunity may play a role
Mekaru et al. (174)	*in vivo* human female	Retrospective study	Evaluation of whether insulin resistance in patients without PCOS affects the results of *in vitro* fertilization and pregnancy	There were no differences in response to controlled ovarian hyperstimulation, number of oocytes retrieved, conception rate, pregnancy rate, live birth rate, and incidence of gestational diabetes in women with and without insulin resistance	Insulin resistance in patients without PCOS has no impact on IVF outcomes or perinatal prognosis
Li et al. (175)	*in vivo* human female	Retrospective study	Assessment of the importance of central obesity on the effectiveness of IVF	Women with central obesity had significantly more endocrine and metabolic disorders and required significantly higher doses of gonadotropins, longer duration of ovarian stimulation	Central obesity negatively affects the effectiveness of IVF
Issa et al. (176)	*in vivo* human female	Case study	Case presentation	Severe hypertriglyceridemia and secondary acute pancreatitis and diabetic ketoacidosis have occurred following *in vitro* fertilization	IVF may be associated with hypertriglyceridemia with secondary acute pancreatitis
Vuguin et al. (177)	*in vivo* animal (mouse) female	Animal medical experiment	Assessment of the importance of glucagon in the regulation of fetal growth and maturation	Deletion of the GLU receptor negatively affected fetal survival and in adult animals resulted in a change in the β/α cell ratio	Glucagon plays an important role in embryogenesis
Saleh et al. (178)	*in vivo* human male	Comparative study	Assessment of the importance of insulin resistance and irisin concentration in the etiology of male idiopathic infertility	Infertile patients had higher HOMA-IR and lower irisin compared to controls	Insulin resistance is one of the potential factors of idiopathic male infertility
Izzi-Engbeaya et al. (179)	*in vivo* human male	Randomized controlled trial	Evaluation of the effect of glucagon administration on the secretion of sex hormones in healthy young men	There were no significant differences in circulating LH, FSH or testosterone levels, although glucagon administration had a metabolic effect	Intravenous administration of glucagon does not affect the secretion of sex hormones in healthy men

### 7.1 Female

#### 7.1.1 *In vivo* human studies

Glucagon is a central mediator of glycemic control, released by pancreatic alpha cells in response to hypoglycemia. It is involved in insulin signaling and action, but also predisposes to adverse pregnancy outcomes. Interestingly, more and more reports confirm the modulating activity of glucagon-like peptide-1 (GLP-1) in reproduction. GLP-1 receptor antagonists play an important role in the treatment of type 2 diabetes and obesity by lowering glucose levels, reducing body weight and improving reproductive health. Obesity inhibits the hypothalamic-pituitary-gonadal axis, disrupting ovarian function, ovulation index and endometrial receptivity, and affects the molecular mechanisms that regulate the biological activity of the reproductive system (3, 181, 182).

Pancreatitis accompanying pregnancy is not common, but it carries a burden in terms of maternal mortality or perinatal mortality (183). Commonly used in hormone contraception, hormone replacement therapy (after menopause) or hormone therapy in *in vitro* fertilization (development of the endometrium in preparation for embryo transfer), estrogens may be one of the causes of acute drug-induced pancreatitis (184). These cases are rare and the clinical course is usually mild to moderate (185). The mechanism of acute estrogen-induced pancreatitis is not fully understood, but it is presumed to be due to hypertriglyceridemia. Mechanisms related to this phenomenon include an increase in the synthesis of TG in the liver, secretion of VLDL into the circulation, an increase in newly synthesized TG in the liver, excessive secretion of TG and apolipoprotein, and the effect of estrogens on the inhibition of lipoprotein lipase (LpL) promoter activity (176). Transported by chylomicrons and very low-density lipoproteins, triglycerides are hydrolyzed by high lipase concentrations in the pancreatic capillaries, where they form a large amount of toxic free fatty acids that cause lipotoxicity in acute pancreatitis (186). Hence, in the case of patients qualified for IVF, especially those from the high-risk group (such as diabetes, polycystic ovary syndrome, obesity, dyslipidemia), it is reasonable to screen for lipid abnormalities before starting the *in vitro* procedure and constantly monitor key biochemical parameters (176).

#### 7.1.2 *In vivo* animal studies

Studies in a mouse model (glucagon receptor knockout mice) showed that the lack of glucagon signaling did not alter the hypothalamic-pituitary-ovarian axis. Pregnant knockout female mice exhibited hypoglycemia and hyperglucagonemia accompanied by decreased fetal weight, increased late-stage fetal mortality, and placental abnormalities. Moreover, the lack of glucagon signaling significantly reduced the level of expression of genes controlling growth, adrenergic signaling, vascularization, oxidative stress and the activity of G-protein-coupled receptors (187). Studies on an animal model allow the conclusion that, similar to insulin, glucagon contributes to the proper reproductive function of women. It has also been shown that the lack of glucagon signaling creates a poor quality uterine environment for fetal growth. Animal studies have demonstrated that the lack of glucagon signaling during pregnancy was associated with reduced litter size, limited intrauterine growth, and increased neonatal mortality (177).

### 7.2 Male

#### 7.2.1 *In vivo* human studies

In a study of male patients with idiopathic infertility, the relationship of metabolic syndrome, obesity and diabetes mellitus was shown as factors contributing to the pathogenesis of male infertility. A strong association of insulin resistance has also been shown in patients with unexplained fertility problems, presuming that elevated blood insulin levels may impair spermatogenesis, and in patients with hyperinsulinemia and type 2 diabetes, also generate nuclear and mitochondrial DNA damage in sperm (178, 188). In the aspect of metabolic diseases, the relationship between sperm count and BMI was also investigated and it was shown that obese men had a twofold increase in the risk of oligozoospermia and increased scrotal temperature, leading to sperm dysfunction, reduced sperm count and mobility, and DNA damage (188).

Moreover, it has been confirmed that glucagon can directly stimulate the reproductive axis, which has not been observed in studies on men where there was no effect of glucagon administration on reproductive hormone levels (179). On the other hand, administration of glucagon receptor antagonists can cause metabolic effects (weight loss, i.e. alleviation of hypogonadism of the hypothalamus in obese men) without direct negative effects on the reproductive system (179).

## 8 Adrenal glands

Changes in the hypothalamic-pituitary-adrenal axis and the subsequent alterations in the concentration of circulating hormones constitute the body’s response to stressful challenges (**Table 6**). Mobilization of resources during the stress response suppresses the reproductive axis, which gives the survival of an individual higher priority than the preservation of the species (195). Stressors also affect the adrenal medulla, which secretes catecholamines, i.e. adrenaline and noradrenaline, and the adrenal cortex that secretes aldosterone, which is, next to cortisol, a key regulator of blood pressure (196).

**Table 6:** Adrenal-related fertility studies.

Authors (reference)	Type of study	Study design	Aim	Results	Conclusion
Csemiczky et al. (189)	*in vivo* human female	Comparative study	Assessment of prolactin and cortisol levels as well as assessment of the personality profile of infertile women	Infertile women also had significantly higher levels of prolactin and cortisol throughout their menstrual cycle	Infertile women have higher serum cortisol and anxiety levels
Smeenk et al. (190)	*in vivo* human female	Multicenter study	Evaluation of the relationships between the concentrations of stress hormones in the urine: adrenaline, noradrenaline and cortisol in women entering IVF for the first time	A significantly higher concentration of the assessed hormones was found before IVF, which decreased in the case of IVF success	Cortisol is the missing link in the relationship between psychosocial stress and outcome after IVF/ICSI
Piquer et al. (191)	*in vivo* animal (rat) female	Animal medical experiment	Determination of changes in the expression of the placental norepinephrine transporter during pregnancy and their relationship with its ability to transport norepinephrine under stress conditions	Exposure of pregnant rats to sympathetic stress resulted in increased levels of norepinephrine and corticosterone throughout pregnancy, decreased placental capacity to clear fetal norepinephrine into the maternal circulation, altered levels of placental epinephrine transporter protein depending on fetal sex, and increased placental and offspring body weight	Increased placental adrenaline transporter levels in pregnancy have been associated with decreased adult fertility of offspring
Kapoor et al. (192)	*in vivo* animal (guinea pigs) female	Animal medical experiment	Assessment of the impact of prenatal stress on the function of the hypothalamic-pituitary-adrenal (HPA) axis in adult offspring	The concentration of cortisol in the offspring of mothers exposed to stress was significantly higher than in the control	Stress exerted on the pregnant female during neuroendocrine development programs growth, HPA axis function and stress-related behavior in adult male guinea pigs
Mayerhofer et al. (193)	*in vivo* animal (hamster) male	Animal medical experiment	Evaluation of the effect of catecholamines on androgen production during periods of gonadal activity and rest in a seasonally reproducing species	Catecholamines have been shown to modulate the Leydig cell response to gonadotropins in this species of hamster	Stress hormones have a negative impact on reproductive functions and abilities
Rehman et al. (194)	*in vivo* human male	Retrospective study	Comparing the concentration of stress markers and antioxidants in fertile and infertile men and examining their impact on reproductive hormones and fertility	Cortisol, epinephrine, follicle-stimulating hormone and luteinizing superoxide dismutase, glutathione levels were significantly higher in the group of patients compared to the control group	Stress together with the reduction of antioxidant concentration plays an important role in reducing the reproductive potential in infertile men

### 8.1 Female

#### 8.1.1 *In vivo* human studies

Past studies have shown that patients with lower levels of adrenaline had a higher rate of implantation (the group of patients undergoing IVF), which suggests that the less stressful life they lead, the greater their reproductive success (189, 190). It has been found that stressors activate the hypothalamic-pituitary-adrenal axis, causing changes that have a significant impact on female fertility (197).

The IVF procedure itself may be a stressful condition. In a study by Smeenk et al. there was no difference in the stress response between women who became pregnant after treatment and those who did not. Little is known about the effects of catecholamines and cortisol on the physiological processes related to reproduction. It is concluded that catecholamines may affect fertility by altering uterine blood flow, while cortisol with immunosuppressive properties may additionally affect the immune states necessary for implantation. Studies have shown that a higher ratio of serum cortisol to follicular cortisol was associated with pregnancy, and that infertile women had higher levels of stress in terms of circulating prolactin and cortisol compared to fertile women in the control group (190).

Aldosterone, as a mineralocorticoid hormone secreted by the zona glomerulosa of the adrenal cortex, is responsible for the regulation of water and electrolyte balance and blood pressure. During physiological pregnancy, aldosterone levels increase, inducing elevated plasma volume, which is essential for the maintenance of circulating blood volume, blood pressure, and uteroplacental perfusion. Aldosterone levels remain high throughout pregnancy, suggesting a possible role in the regulation of placental and fetal development (198, 199). Recent studies have shown the possible involvement of aldosterone in some gynecological conditions and diseases, including endometriosis combined with infertility. Aldosterone may exacerbate systemic and local inflammatory states underlying endometriosis by activating mineralocorticoid receptors present in inflammatory cells (198).

#### 8.1.2 *In vivo* animal studies

High levels of the norepinephrine transporter (NET) have been documented in mammalian placenta tissue. Placental changes in NET were involved in the modification of reproductive function and fertility of the offspring caused by pregnancy stress (191). Studies in rats showed that exposure of pregnant rats to sympathetic stress affected the placental transport of norepinephrine, leading to a reduction in the ability of the placenta to remove norepinephrine from the fetus into the maternal circulation. It also resulted in impaired fertility of the offspring in adulthood (191). In addition, it has been observed that increased plasma levels of norepinephrine can result in the narrowing of the uterine arteries leading to failure of trophoblast invasion.

Pregnancy is particularly sensitive to stress factors that may lead to permanent modifications in the postpartum development of newborns, predisposing them to diseases that appear in adulthood (200, 201). In a guinea pig study, maternal access to nutrients was drastically restricted during the period of maximum fetal brain growth (70% of pregnancy), resulting in adult male offspring with elevated basal cortisol levels but normal adrenal stress response. However, the same stress applied to 90% of pregnancy results in male offspring with normal basal cortisol levels but increased responsiveness of the hypothalamic-pituitary-adrenal axis to challenge (192). Interspecies differences in the effects of prenatal stress on adult behavior may also be due to the unique development profiles of fetal bodies and brains. In humans, the rapid phase of fetal brain development occurs from about 27 to 30 weeks of gestation and extends to the postpartum period, while in rats or mice, maximum brain growth does not begin until postnatal life. Hence, the period of maternal stress in a rodent would probably correspond to a completely different phase in the human fetal brain.

### 8.2 Male

#### 8.2.1 *In vivo* human studies

Apart from oxidative stress, which has a negative impact on the quality of sperm, long-term stress manifested by an increased level of cortisol is also mentioned. This is related to male infertility due to the decreased conversion of androstenedione to testosterone, thereby reducing the volume and concentration of sperm in semen (202). Studies have shown that cortisol and adrenaline levels were significantly higher in the group of infertile men compared to the control group of men without fertility problems (194). Stress in this sense is both a physical and an emotional factor causing the activation of neurons that secrete the corticotropin-releasing hormone, leading to higher plasma cortisol levels. Excess cortisol reduces testosterone production, reducing sperm parameters. Cortisol may also directly reduce testosterone production by blocking the transcription of genes encoding enzymes necessary for testosterone synthesis (195).

#### 8.2.2 *In vivo* animal studies

High levels of adrenaline and associated low testosterone have also been observed in infertile golden hamsters (193). This phenomenon, also accompanying short-term stresses, results from the inhibitory effect of adrenaline and dopamine on testosterone production both by the HPA hypothalamic-pituitary axis and the peripheral blockade of testosterone release (203).

## 9 Ovaries

Estrogens belong to the family of steroid hormones produced mainly by the gonads and the placenta. In addition to the reproductive system, they play an important role in the immune, skeletal and neuroendocrine systems, therefore disturbances in their concentration or functioning are observed in pathological conditions and diseases such as infertility, cancer, obesity or osteoporosis (204). Estrogens have been shown to have negative and positive feedback on the hypothalamic-pituitary axis, and their action is possible by binding to the estrogen receptor alpha (ERα) and beta (ERβ). These receptors have different tissue expression patterns in both humans and rodents (205). Estrogen mediation in biological responses is possible through a genomic mechanism, usually occurring within hours in most tissues, and a non-genomic mechanism, occurring very rapidly within minutes of exposure to hormones (206).

Estrogen binds to the ER, which is mainly found in the nucleus of the target cell. The resulting complex can regulate gene activity by binding directly to DNA regulatory elements called estrogen response elements (EREs), leading to the recruitment of additional factors involved in the regulation of transcription (207). An alternative approach, also called indirect, involves interaction with transcription factors, including TF, AP-1, SP1, and NF-κB, which recruits chromatin-modifying coregulator proteins and enables activation or repression of ER target genes to direct cell proliferation (208). The non-genomic mechanism involves the interaction of the ER located in the plasma membrane or its vicinity with adapter proteins, including Shc and Src, as well as signaling through GPR30, resulting in activation of the MAPK cascades, PI3K, and adenylate cyclase (209).

Progesterone is an endogenous steroid hormone commonly produced by the cortex of the adrenal glands and the gonads. The ovarian follicles are the main source of peripheral progesterone from the late follicular to luteal phase (210). It is also secreted by the corpus luteum of the ovary, generally during the first ten weeks of pregnancy, and then through the placenta (211). The action of progesterone is based on binding to a receptor located in the cell cytoplasm. Then it dimerizes and translocates to the nucleus where it can bind to DNA, which enables the regulation of gene expression. There are three isoforms of progesterone receptors: PR-A, PR-B and PR-C (212).

### 9.1 Primary ovarian insufficiency

Primary ovarian insufficiency (POI) is defined as the cessation or irregular menstrual cycles under the age of 40 in the presence of elevated serum FSH levels. This disorder is rare and affects approximately 1% of women. The causes of POIs can be spontaneous, genetic, environmental, infectious, autoimmune, surgical, chemotherapy or radiation related (213). The European Society of Human Reproduction and Embryology (ESHRE) recommends both of the following diagnostic criteria for POIs: hypomenorrhea/amenorrhea for at least four months, elevated follicle-stimulating hormone levels (> 25 mIU/mL) confirmed twice at an interval > 4 weeks (214). Serum FSH determination is the gold standard in the POI diagnosis. The anti-müllerian hormone can only be interpreted in conjunction with the FSH and estrogen levels. In the case of secondary amenorrhea, it is necessary to exclude pregnancy by testing the serum level of beta subunit of human chorionic gonadotropin (beta-HCG) and the concentration of thyroid stimulating hormone and prolactin, as endocrine diseases can lead to menstrual disorders (215). Chromosome analysis and fragile X premutation tests are recommended for all women with POI (216). As some cases of POI are autoimmune in nature, it is important to rule out other autoimmune diseases. According to ESHRE, it is necessary to assess the level of adrenal and thyroid antibodies. If the results are positive, it is imperative to monitor the function of these glands (214).

About 76% of POI patients maintain regular periods during adolescence and adulthood, followed by cycle disruptions (217). Ovarian function can be intermittent and unpredictable, with spontaneous ovulation in up to 20% and conception in approximately 5-10% of women (163). The symptoms these women experience are identical to those during menopause and can include hot flashes, night sweats, dyspareunia, vaginal dryness, sleep disturbances, mood changes, altered urination frequency, low libido, and a lack of energy. They are caused by a decrease in the production of estradiol in the ovaries. Symptoms may be transient or intermittent and may vary in severity due to fluctuations in ovarian activity during the spontaneous occurrence of POI (218). POI significantly reduces patients’ quality of life due to increased cardiovascular risk, decreased bone mineral density leading to osteoporosis and atrophic changes in the genitourinary system. The disease has a negative impact on the mental well-being of patients, is associated with pregnancy failures and lower sexual satisfaction (219).

### 9.2 Polycystic ovary syndrome

Polycystic ovary syndrome (PCOS) is the most common endocrine disorder in women of childbearing age, affecting approximately 6–21% of women (220). PCOS can be diagnosed using the Rotterdam criteria, which require at least two of the three listed criteria: anovulation, clinical signs of hyperandrogenism and/or serological elevation of androgens, polycystic ovaries demonstrated by ultrasound. The National Institutes of Health criteria also require clinical or biochemical hyperandrogenism and oligo- or anovulation. The American Excess PCOS Society requires hyperandrogenism with one of the other two criteria. As this is an exclusion diagnosis, disorders that exhibit PCOS-like features must be ruled out. These include hyperprolactinemia, thyroid disease, non-classical congenital adrenal hyperplasia (221, 222).

In addition to infertility, many diseases are associated with PCOS, including endometrial cancer, type 2 diabetes, impaired glucose tolerance, metabolic syndrome, cardiovascular risk, non-alcoholic fatty liver disease/non-alcoholic steatohepatitis (NAFLD/NASH) (223). It has been estimated that about 50% of women with PCOS are overweight or obese and have reduced insulin sensitivity (224). Almost all causes of PCOS arise from functional ovarian hyperandrogenism (FOH). Two-thirds of cases have typical FOH, characterized by androgen secretion disruption with an excessive response of 17-hydroxyprogesterone to gonadotrophin stimulation. In other cases of PCOS, an increase in testosterone levels is observed, which can be detected after suppressing the production of androgens in the adrenal glands (225). The causes of dysregulation include excess insulin, which sensitizes the ovaries to luteinizing hormone, as well as an imbalance between the intra-ovarian regulatory systems.

The excess of androgens enhances the growth of primary follicles. At the same time, it initiates premature luteinization, making the selection of the dominant follicle difficult (226). It is estimated that about half of FOH patients have insulin-resistant hyperinsulinism, which prematurely luteinizes granulosa cells, increases steroidogenesis, and stimulates fat accumulation. Hyperandrogenemia causes LH excess, which acts on luteinized granulosa and theca cells (227). Hormonal dysregulation alters the pulsatile release of GnRH, resulting in increased biosynthesis and secretion of LH compared to FSH (228). It has been shown that LH stimulates the production of androgens in the ovaries, while a decrease in FSH levels leads to the inhibition of aromatase activity in granulosa cells, reducing the conversion of androgens to estradiol (229). Moreover, serum androgens are converted peripherally to estrogens. Since it occurs mainly in adipose tissue, estrogen production will be increased in obese PCOS patients. Furthermore, unbalanced estrogen stimulation can lead to endometrial hyperplasia and endometrial cancer (230).

### 9.3 Female

#### 9.3.1 *In vivo* human studies

Estrogens, in particular estradiol-17β, are essential fertility regulating hormones. This is related to their participation in the development of ovulatory follicles, induction of pre-ovulatory gonadotropin release in the middle of the cycle, or preparation of the uterine mucosa for implantation. Changes in the production and/or function of estrogen can therefore disrupt these processes, leading to infertility (207) (**Table 7**). Studies carried out so far in models using rats have shown that disruption of Esr1 causes infertility in both males and females (231). Rumi et al. also concluded that the proper functioning of Esr2 is the primary regulator of female fertility, but is not critical to male fertility (232).

**Table 7:** Ovarian-related fertility studies.

Authors (reference)	Type of study	Study design	Aim	Results	Conclusion
Rumi et al. (231)	*In vivo* animal (rat) female	Animal medical experiment	Analysis of Esr1-knockout rats generated by ZFN-mediated genome editing	Δ482 *Esr1* mutation created a null allele	Disturbance of ESR1 activity leads to infertility of male and female rats
Rumi et al. (232)	*In vivo* animal (rat) female	Animal medical experiment	Assessment of the role of ESR2 in fertility regulation	Reproductive functions were impaired in mutant rats	Disturbance of ESR2 activity leads to female infertility
Hipp et al. (233)	*in vivo* human female	Semi-structured interviews	Analysis of diagnostic and therapeutic regimens in people with fragile X-associated POI	POI diagnosis and hormone treatment occurred later	Women with FXPOI are at risk for delayed POI diagnosis and undertreatment with hormone therapy
Mohammed et al. (234)	*in vivo* human female	Meta-analysis	Risk comparison of vascular events during the use of oral and transdermal estrogen therapy in postmenopausal women	A higher percentage of vascular events occurred in women using oral estrogens	Oral estrogen therapy may be associated with a higher risk of vascular events compared to transdermal therapy
Canonico et al. (235)	*in vivo* human female	Clinical experiment	Assessment of the impact of HRT administration on the risk of venous thromboembolism	The odds ratio for venous thromboembolism was higher in women using oral estrogens	Transdermal estrogen therapy is associated with a lower risk of venous thromboembolism
Renoux et al. (235)	*in vivo* human female	Comparative study	Assessment of the impact of HRT administration on the stroke risk	A higher stroke rate was reported in the oral HRT group	Oral HRT may increase the risk of stroke
Langrish et al. (236)	*in vivo* human female	Randomized controlled trial	Evaluation of HRT effects in women with POI	Better kidney function and lower blood pressure have been demonstrated for the physiological substitution of sex steroids	Physiological sex steroid substitution is a better option for young women with POI
Piedade et al. (237)	*in vivo* human female	Observational study (case report)	Analysis of the therapeutic regimen in women with POI	The patient became pregnant and gave birth to a healthy child	Actions should be taken to select the optimal treatment method
Pinelli et al. (238)	*in vivo* human female	Retrospective analysis	Analysis of estrogen treatment regimen in women with POI	FSH levels were lower and the number of collected and fertilized MII oocytes was higher	Estrogen treatment facilitates conception and improves IVF outcomes
Zhang et al. (239)	*in vivo* human female	Meta-analysis	Assessment of the impact of a low-carbohydrate diet on the clinical symptoms of PCOS	A low-carbohydrate diet helps to improve the outcomes of PCOS patients	Proper control of carbohydrate intake can alleviate the symptoms of PCOS
Glintborg et al. (240)	*in vivo* human female	Randomized controlled trial	Evaluation of the effects of metformin use in patients with PCOS	The use of metformin promotes weight loss and improves body composition	Treatment with metformin in combination with oral contraceptive pills is preferable to treatment with the pill alone
Takasaki et al. (241)	*in vivo* human female	Clinical experiment	Assessment of the effectiveness of modification of standard clomiphene treatment in patients resistant to this drug	About 80% of patients had positive results	Intermittent clomiphene treatment is a useful option in people who are resistant to this drug
Homburg et al. (242)	*in vivo* human female	Randomized controlled trial	Comparison of the effectiveness of clomiphene and FSH treatment in patients with PCOS	A higher percentage of pregnancies and live births after using FSH instead of clomiphene has been shown	FSH may be an appropriate first-line therapy for some women with PCOS
Waanbah et al. (243)	*in vivo* human female	Observational study (cohort study)	Comparison of the effectiveness of clomiphene and letrozole treatment in patients with PCOS	A higher percentage of pregnancies and healthy births was observed after treatment with letrozole	Letrozole is a better ovulation inducer than clomiphene in women with PCOS
Ganie et al. (244)	*in vivo* human female	Randomized controlled trial	Assessment of the effectiveness of spironolactone and metformin treatment in patients with PCOS	Combination treatment gave better results	A low dose of spironolactone and metformin may have beneficial effects in the treatment of PCOS
Glintborg et al. (245)	*in vivo* human female	Randomized controlled trial	Assessment of the effect of oral contraceptives and metformin on GLP-1 secretion in patients with PCOS	GLP-1 levels increased during treatment	The use of oral contraceptives and metformin affects the secretion of GLP-1
Devin et al. (246)	*in vivo* human female	Randomized controlled trial	Assessment of the relationship between the levels of growth hormone and sitagliptin and its impact on visceral obesity in patients with PCOS	Sitagliptin lowered visceral fat levels and increased growth hormone half-life and pulse spacing	Sitagliptin may be useful in the treatment of PCOS in obese women
Javed et al. (247)	*in vivo* human female	Randomized controlled trial	Assessment of the effect of empagliflozin on the metabolic parameters of patients with PCOS	Anthropometric parameters and body composition improved after treatment with empagliflozin	Empagliflozin may be useful in the treatment of PCOS in obese women
Benito et al. (248)	*in vivo* human female	Observational study (cohort study)	Assessment of the fertility level in women with PCOS after bariatric surgery	Pregnancy and fertility rates were high after surgery and there were few maternal and neonatal complications	Bariatric surgery has a positive effect on pregnancy and fertility indicators
Toulis et al. (249)	*in vivo* human female	Meta-analysis	Risk assessment of gestational diabetes in women with PCOS	There is a possible risk of gestational diabetes in women with PCOS	PCOS may be a potential risk factor for the development of gestational diabetes
De Frène et al. (250)	*in vivo* human female	Comparative study	Assessment of the influence of overweight and PCOS on the risk of perinatal complications	Women with PCOS and who were overweight had an increased risk of perinatal complications	Weight loss before pregnancy can reduce the risk of perinatal complications

#### 9.3.2 *In vivo* animal studies

Physiological estrogen replacement alleviates menopausal symptoms and may improve sexual dysfunction associated with vaginal dryness, dyspareunia, and decreased libido (251). Unfortunately, Hipp et al. showed that more than half of young women with POI either never use hormone replacement therapy (HRT) or start it many years after diagnosis and/or stop using it before the age of 45 (233). The current evidence supports transdermal or vaginal therapy with estradiol as the first line of HRT. They mimic the daily rate of ovarian estradiol production and achieve mean serum level of 100 pg/mL, which is the average level in women with normal ovarian function throughout the menstrual cycle (252). Oral estradiol may be an alternative, but this is associated with complications related to the hepatic first-pass effect, including increasing the risk of venous thromboembolism (234). The multicenter study of Estrogen and Thromboembolism Risk (ESTHER) confirmed that the odds ratio for this disease was 4.2 in oral estrogen users compared to 0.9 in transdermal estrogen users (235). Interestingly, Renoux et al. also noted that the use of oral HRT was associated with a higher rate of stroke in postmenopausal women (253). In addition, Langrish et al. conducted a study in which they compared the treatment effects of transdermal estradiol and cyclic progestins with treatment with combined oral contraceptives on the circulatory system of young women with POI. They noticed that 12-month transdermal HRT resulted in significantly lower blood pressure, better kidney function and reduced activation of the renin-angiotensin-aldosterone system, suggesting that it was more effective (236).

More and more studies reveal that reproductive disorders can have a negative impact on pregnancy, from implantation to delivery. Many patients with these conditions require assisted reproductive technology (ART), which may influence pregnancy outcomes. As a result, it is difficult to distinguish the contribution of certain factors to poor pregnancy outcomes (254). It has been shown that often multiple risk factors contribute to negative obstetric outcomes in women with reproductive disorders. In addition, many women of childbearing age have more than one reproductive disorder (255).

In the case of POI, the chances of a spontaneous pregnancy are very small. According to the ESHRE guidelines, there are no interventions that increase the chances of natural conception, so oocyte donation or reception is considered a reliable chance to become pregnant (214). Interestingly, most people with POI still have detectable follicles. The mechanism of follicle dysfunction was defined in the NIH study as its inappropriate luteinization due to tonically increased serum LH levels. HRT that suppress high LH levels, can increase the chances of ovulation and improve follicle function. Piedade et al. presented a case report where a patient chose intra-uterine insemination (IUI) in combination with follicle monitoring and HRT and successfully became pregnant (237). In turn, Pinelli et al. conducted a study in which women with reduced ovarian reserve were administered valerate estradiol (2 mg daily) adding dihydrogesterone (10 mg daily) in the luteal phase for 3 months prior to the standard short protocol with a GnRH antagonist for *in vitro* fertilization. They concluded that pretreatment with estrogen appeared to improve IVF outcomes (238).

For overweight and obese women with PCOS, exercise and calorie restriction diets are the best first-line interventions for weight loss and impaired glucose tolerance (IGT) reduction. It is possible to alleviate the clinical symptoms of PCOS, including abnormal ovulation and hormone disturbances. Zhang et al. in their meta-analysis assessed the effectiveness of a low-carbohydrate diet in the treatment of PCOS and showed its beneficial effects (239). Treatments for menstrual disorders, hirsutism and acne include oral contraceptives, transdermal patch or vaginal ring. The progestogen component lowers LH levels, indirectly reducing the production of androgens in the ovaries and increasing the level of sex hormone binding globulin. Metformin is recommended in case of contraindications to the use of hormonal contraceptives. It has been shown to reduce the progression from impaired glucose tolerance to type 2 diabetes and also to improve menstrual cycles and vascular markers in non-obese women with PCOS (240).

Clomiphene citrate is considered the first-line treatment for infertility in PCOS patients. It is a selective estrogen receptor modulator (SERM) that is a competitive inhibitor of estrogen receptors. Clomiphene improves fertility and ovulation, especially by acting on the hypothalamus, where it binds to estrogen receptors. This causes a pulsatile release of GnRH, promoting the secretion of gonadotrophins from the anterior pituitary. Interestingly, up to 25% of women do not respond to the administration of clomiphene, which is associated with the implementation of gonadotropin therapy. However, Takasaki et al. proposed to modify the clomiphene treatment regimen. They used intermittent dosing, which was effective in approximately 80% of clomiphene-resistant patients (241). On the other hand, Homburg et al. showed that a small dose of FSH could be used instead of clomiphene (242). Another first-line drug for ovulation induction is letrozole. It is an aromatase inhibitor that blocks estrogen synthesis, reducing negative estrogen feedback in the pituitary gland. Waanbah et al. showed that treatment with letrozole, compared to clomiphene, was associated with higher rates of live births and ovulation among infertile women with polycystic ovary syndrome (243). Spironolactone, which is an antagonist of the mineralocorticoid receptor, is also used, as well as its combinations with metformin, which helped to improve the menstrual cycle, glucose and testosterone levels (244).

In PCOS patients, insulin sensitization is also introduced. It is associated with the administration of glucagon-like peptide 1 (GLP-1) agonists which, upon binding to the receptor, stimulate glucose-dependent insulin release from the pancreatic islets. This treatment reduced BMI and testosterone, and improved the ovulation rate in obese women with PCOS (256). Glintborg et al. showed that after treatment with oral contraceptives along with metformin, the level of GLP-1 in women with PCOS was comparable to that in healthy people (245). In addition, Devin et al. conducted studies using DPP4 inhibitors that have beneficial effects on weight loss and lowering blood glucose levels in obese women with PCOS. They reported that the effect of DPP4 inhibitors on the body weight of women with PCOS is based on the increase in the growth hormone levels, which are lowered in PCOS patients. This, in turn, reduces the mass of visceral adipose tissue (246). On the other hand, Javed et al. noted the promising effect of SGLT2 inhibitors in overweight and obese women with PCOS. After 12 weeks of treatment with empagliflozin, there was an improvement in body composition and anthropometric parameters compared to metformin treatment, however no changes in hormonal and metabolic parameters were observed (247). Interestingly, Benito et al. conducted a study in women with POCS who had undergone bariatric surgery. They observed a significant improvement in pregnancy and healthy birth rates compared to the control group (248). In turn, Toulis et al. showed that up to 40% of women with PCOS and glucose intolerance can develop gestational diabetes (GDM) (249). There was also an increased risk of pregnancy and neonatal complications, including premature pregnancy loss, gestational hypertension, preterm delivery, low birth weight and the need for cesarean delivery, regardless of obesity (250).

## 10 Testes

The testes are paired, oval glands located in the scrotum. Their main functions include the production of male gametes (sperm) and male sex hormones. Sperm formation is carried out by the germinal epithelium located in the convoluted tubules of the testes, while the production of testosterone, the main male sex hormone, is the responsibility of Leydig cells. These cells also produce a small amount of androstenedione (A4) (257). The functioning of the testes depends mainly on LH and FSH, as LH stimulates the production of testosterone, and FSH promotes the production of male gametes (258).

The testes are not only the site of testosterone synthesis, but also the main organ regulated by its activity. Testosterone stimulates spermatogenesis by affecting not only spermatogonial stem cells, but also Sertoli cells, which support spermatogenesis and create a blood-testis barrier within the epithelium (259). In addition, testosterone is the main hormone involved in male sexual maturation (260). Other functions of testosterone are the stimulation of bone and muscle mass growth, and the regulation of sexual drive (261). Unlike LH, FSH acts directly on epithelial cells. It regulates the function of Sertoli cells, stimulating them to paracrine activity, promoting the maturation of spermatogonia into spermatocytes (262). The large increase in testosterone production by the testes in adolescence is one of the main factors in stimulating puberty. During sexual maturation, its synthesis increases 20-30 times (263). Due to this, not only physical but also mental changes are induced, such as increased sex drive, tendencies towards non-physical aggressive behavior and domination (264).

The testes, although being the main androgen-producing organ, are not the only ones. Another source of androgens is the adrenal glands. They produce dehydroepiandrosterone (DHEA) and androstenedione (265). DHEA is an anabolic hormone and strongly influences cognitive functions such as mood and sex drive (266). A4 is a testosterone precursor and has an effect similar to testosterone, but weaker. Both hormones are involved in adrenarche, and the increase in their concentration at the age of 6-8 years is probably of key importance in the development of social and cultural skills (267). Impaired testosterone secretion, regardless of whether it results from primary organ failure (Klinefelter syndrome, Leydig cell aplasia, genetic or drug-induced defects) or a defect associated with the secretion of pituitary hormones (Kallmann’s syndrome, hypopituitarism, genetic or drug-induced defects in LH secretion) is a well-known cause of male infertility (268) (**Table 8**).

**Table 8:** Male fertility studies.

Authors (reference)	Type of study	Study design	Aim	Results	Conclusion
Mohanty et al. (269)	*in vivo* human male	Bioinformatic analysis of available databases	Assessment of the effect of HSPA2 polymorphism on male fertility	The existence of a variant of the HSPA2 gene negatively affecting fertility was demonstrated	HSPA2 polymorphism may cause male infertility
Choobineh et al. (270)	*in vivo* human male	Clinical experiment	Assessment of the effect of testosterone administration on the reduction of the negative impact on the testes after spinal cord injury	Increased semen quality in the study group was demonstrated	Early administration of testosterone after a spinal cord injury may positively influence fertility preservation
Snyder et al. (271)	*in vivo* animal male	Animal medical experiment (mouse)	Analysis of the effect of ADAD1 and ADAD2 expression on male fertility	Mice with blocked expression of ADAD1 and ADAD2 were completely sterile	ADAD1 and ADAD2 are key genes related to fertility
Chi et al. (272)	*in vivo* animal male	Animal medical experiment (mouse)	Assessment of the effect of Kindlin-2 expression on male fertility	Testicular hypoplasia and male infertility have been observed after Kindlin-2 expression was blocked	Kindlin-2 is a key gene related to fertility
Wei et al. (273)	*in vivo* animal male	Animal medical experiment (mouse)	Establishing the mechanism of reducing fertility in males with the Wip1 phosphatase mutation	Changes in the expression of numerous proteins related to cell adherence, apoptosis, response to the stimulation of pro-inflammatory cytokines and spermatogenesis were detected	Disabling Wip1 alters the expression of numerous fertility-related genes
Xiang et al. (274)	*in vivo* animal male	Animal medical experiment (mouse)	Assessment of the effect of Crybb2 expression on male fertility	After blocking Crybb2, fertility decreased significantly	Crybb2 is one of the key fertility genes
Sun et al. (275)	*in vivo* animal male	Animal medical experiment (mouse)	Searching for genes important for male reproduction using the CRISP method	A significant role of 13 genes in spermatogenesis and sperm maturation was determined	Genetic mutations can directly lead to infertility
Xia et al. (276)	*in vivo* animal male	Animal medical experiment (mouse)	Analysis of the effect of testis-expressed protein 33 (TEX33) expression on male fertility	Mice with blocked TEX33 expression showed no impairment of fertility	TEX33 is not related to fertility
Shah et al. (277)	*in vivo* animal male	Animal medical experiment (mouse)	Analysis of the influence of c4orf46 expression on male fertility	Mice with blocked c4orf46 expression showed no impairment of fertility	C4orf46 is not related to fertility
Holcomb et al. (278)	*in vivo* animal male	Animal medical experiment (mouse)	Analysis of the effect of expression of testis-specific serine proteases PRSS44, PRSS46, and PRSS54 on male fertility	Mice with blocked expression of testis-specific serine proteases PRSS44, PRSS46, and PRSS54 showed no impairment of fertility	Testis-specific serine proteases PRSS44, PRSS46, and PRSS54 are probably not related to fertility
He et al. (279)	*in vivo* animal male	Animal medical experiment (mouse)	Assessment of the effect of Trim69 expression on male fertility	No changes in fertility were observed after Trim69 blockade	Trim69 is not a key gene associated with fertility
Xiuying et al. (280)	*in vivo* animal male	Animal medical experiment (Rana nigromaculata)	Assessment of the effect of microcystin-leucine-arginine on reproduction	Decrease in fsh synthesis after mclr administration	Exposure to mclrs negatively affects fertility
Yang et al. (281)	*in vivo* animal male	Animal medical experiment (mouse)	Assessment of the effect of T-2 toxin on male fertility	The administration of T-2 toxin caused a significant decrease in the concentration of hormones of the hypothalamic-pituitary-testis axis, which reduced fertility	Exposure to T-2 toxin negatively affects fertility
Zhang et al. (282)	*in vivo* animal male	Animal medical experiment (rat)	Assessment of the effect of exposure to Mn_3_O_4_ nanoparticles on male fertility	The administration of Mn_3_O_4_ nanoparticles caused damage to the testes and decreased fertility	Exposure to Mn_3_O_4_ nanoparticles negatively affects fertility
Domínguez-Salazar et al. (283)	*in vivo* animal male	Animal medical experiment (rat)	Analysis of the effect of sleep deprivation on the blood-testicular barrier in males	In rats subjected to sleep deprivation, a decrease in the expression of proteins that build the blood-testicular barrier was observed, which leads to reduced fertility; fertility functions return to normal after sleep deprivation is reversed	Sleep deprivation has a negative effect on fertility, however it is reversible
Ye et al. (284)	*in vivo* animal male	Animal medical experiment (mouse)	Assessment of the effect of metformin administration in obese males on fertility	Increased fertility in the study group was demonstrated	Metformin can be used to treat male fertility
Akomolafe et al. (285)	*in vivo* animal male	Animal medical experiment (mouse)	Assessment of the effect of star apple fruit on male fertility	Increased fertility in the study group was demonstrated	Star apple fruit can be used as a nutritional supplement to improve fertility
Silva et al. (286)	*in vitro* human male	Bioinformatics analysis of gene relationships (in men)	Searching for links between amyloid precursor protein (APP) and genes related to fertility	Numerous relationships of APP expression have been determined not only with genes related to spermatogenesis and sperm maturation, but also with genes regulating the interaction of sperm with oocytes	APP expression may be related to fertility
Miyata et al. (287)	*in vivo* animal male	Bioinformatic analysis of available databases	Analysis of potential genes related to male fertility	54 genes potentially unrelated to male fertility were identified	The pool of genes suspected of being associated with male fertility was limited
Lu et al. (288)	*in vivo* animal male	Bioinformatic analysis of available databases	Analysis of potential genes related to male fertility	30 genes potentially unrelated to male fertility were identified	The pool of genes suspected of being associated with male fertility was limited

### 10.1 Male

#### 10.1.1 *In vivo* human studies

The percentage of infertile men ranges from 2.5-12% depending on the studied population (289). Infertile males account for 40-50% of couple infertility, and the trend is increasing (290). The main cause of male infertility is poor semen quality, i.e. changes in physical parameters and volume, a decrease in the number of sperm per unit volume, reduced sperm motility and viability, abnormal sperm morphology. There are many reasons for a decline in sperm quality, from primary and secondary testicular failure to numerous genetic, environmental and lifestyle factors (291).

The genetic factors include not only numerical and structural chromosomal aberrations, but also microdeletions of the Y chromosome (292) and mutations of autosomal genes (293). The genes whose mutations have been associated with the reduction of male fertility include methylenetetrahydrofolate reductase (MTHFR), which is important in DNA methylation in stem cells (294), steroid 5-alpha reductase responsible for the conversion of testosterone into dihydrotestosterone (DHT), i.e. a more active metabolite (295), protamines replacing histone proteins during the condensation of the nuclear chromatin of sperm (296), heat shock protein A2 (HSPA2) responsible for the protection of cells from exposure to stress factors (269).

Environmental and lifestyle factors are mainly related to the induction of oxidative stress (297). Reactive oxygen species disrupt sperm functions and their viability, damaging cell membranes, organelles and genetic material (298). It has been proven that oral administration of antioxidants may be effective in the treatment of some patients with infertility (299).

A positive correlation has also been demonstrated between high plasma testosterone concentration and fertility (300). An important finding is that the administration of exogenous testosterone, which reduces the production of endogenous hormone by suppressing the pituitary gland, correlates with a decrease in male fertility (301). However, it has been proven that early testosterone therapy after spinal cord injury increases the chances of preserving fertility in men (270).

#### 10.1.2 *In vivo* animal studies

Animal studies focus mainly on establishing the relationship of gene expression within the testes with fertility. It has been demonstrated with such genes as ADAD1 and ADAD2 (271), Kindlin-2 (272), Wip 1 (273), and Crybb2 (274). At the same time, the lack of influence on fertility of a dozen or so genes, which are expressed within the testes, has been confirmed (275–279).

Numerous studies have also focused on establishing the effects of various toxins on fertility. It has been shown that exposure to microcystin-leucine-arginine and T-2 toxin suppresses the release of FSH in the pituitary gland, which, by reducing testosterone levels, decreases fertility (280, 281). On the other hand, exposure to mequindox or Mn_3_O_4_ microparticles reduces fertility due to the induction of oxidative stress within the testes (282). Sleep deprivation has a similar effect, with a reversible decline in fertility due to increased oxidative stress (283).

There are also studies related to the treatment of male infertility. The use of metformin in obese males has been shown to increase fertility (284). The effectiveness of supplementation with apple star fruit in order to improve fertility has also been shown (285). It is probably related to the antioxidant activity of both substances (302).

#### 10.1.3 *In vitro* human studies

Currently, *in vitro* research is focused on determining the relationship between gene expression in testicular cells and fertility. The expression of APP, a protein related to the etiology of Alzheimer’s disease, which influences numerous genes directly related to fertility, has been shown to be of great importance (286). Due to bioinformatic analysis, it was possible to determine the potential relationship of 30 genes with fertility and to exclude 54 genes from this group (287, 288).

### 10.2 The relationship between androgens and female fertility

Recently, increasing attention has been paid to the relationship between female fertility and blood levels of androgens (**Table 9**). Their sources are the ovaries, adrenal glands and the conversion of estrogen into androgens in other tissues (310). The concentration of this hormone is not subject to lifestyle fluctuations, except for smoking, which increases its level (311). The causes of the increase in androgen levels in women may include, apart from exogenous supplementation, polycystic ovary syndrome, adrenal hyperplasia, Cushing’s disease and neoplasms (312).

**Table 9:** The relationship of testosterone and female fertility.

Authors (reference)	Type of study	Study design	Aim	Results	Conclusion
Sjaarda et al. (303)	*in vivo* human female	Clinical experiment	Assessment of the effect of testosterone and anti-müllerian hormone levels on conception and pregnancy	In the group of women with a high concentration of testosterone or amh, non-ovulatory cycles are statistically significantly more frequent; the influence of changes in the concentration of these hormones does not affect the course of pregnancy	High levels of testosterone or amh can negatively affect fertility
Lathi et al. (304)	*in vivo* human female	Clinical experiment	Assessment of the effect of testosterone levels on pregnancy in women with PCOS	No statistically significant differences in the course of pregnancy were shown	Testosterone in patients with PCOS does not adversely affect pregnant patients
Valdimarsdottir (305)	*in vivo* human female	Clinical experiment	Assessment of the effect of testosterone levels in the second trimester on pregnancy in women with PCOS and the relationship between testosterone levels and body weight	The higher the body weight, the higher the testosterone concentration; high testosterone levels during the second trimester positively correlate with the risk of preeclampsia	High testosterone levels in pregnant women may result in a higher risk of preeclampsia
Chinnathambi et al. (306)	*in vivo* animal female	Animal medical experiment (rat)	Assessment of the effect of testosterone on the condition of the uterine arteries during pregnancy in a female	A significant increase in the expression of pro-inflammatory factors and changes in the expression of factors determining the tension in the artery wall were demonstrated	Testosterone can negatively affect the vascularization of the uterus in pregnancy
Sun et al. (307)	*in vivo* human female	Clinical experiment	Assessment of the influence of testosterone concentration on the effectiveness of ovarian stimulation and the course of pregnancy achieved by IVF	Low testosterone levels correlate with a poor response to ovarian stimulation, no association with the course of pregnancy has been demonstrated	The use of testosterone during ovarian stimulation may increase the effectiveness of these treatments
Chen et al. (308)	*in vivo* human female	Clinical experiment	Assessment of the influence of testosterone concentration in women on the effectiveness of the ovarian stimulation	Optimal testosterone concentrations have been demonstrated at various stages of the ovarian stimulation	The use of testosterone during the ovarian stimulation process may increase the effectiveness of these treatments
Saharkhiz et al. (309)	*in vivo* human female	Clinical experiment	Assessment of the influence of testosterone administration on ovarian response in IVF cycles	An improvement in ovarian response in women in the study group and a higher pregnancy rate was demonstrated	Testosterone administration in women with a poor ovarian response to stimulation may be effective in IVF

It has been shown that the increased concentration of androgens increases the frequency of anovulatory cycles (303). The effect of increased testosterone concentration on pregnancy is difficult to clearly establish, as both no effect on the course of pregnancy (304) and an increased risk of preeclampsia (305) have been demonstrated. An increased generation of pro-inflammatory factors within the uterine arteries was also observed, which may have a potentially negative impact on the course of pregnancy (306).

At the same time, it has been shown that the reduced testosterone concentration in women undergoing assisted reproductive protocols may be the cause of a poor response to ovarian stimulation (307). Optimal testosterone concentrations have been demonstrated in terms of the effectiveness of assisted reproductive protocols (308) and oral testosterone supplementation in order to increase the chances of success of such treatments (309).

## 11 Summary

More and more couples are affected by fertility and pregnancy maintenance disorders. It is estimated that male infertility accounts for 40-50% of couples’ infertility. It is associated with impaired testosterone secretion, which may result from primary organ failure or is associated with the disruption of the secretion of pituitary hormones. Normal ovarian function and placental secretion are essential for female fertility and the maintenance of pregnancy. The most common endocrine disorder in women of reproductive age is polycystic ovary syndrome, where androgen secretion is impaired, and an increase in testosterone levels is also possible. Such dysregulation results in a disruption of the pulsatile secretion of gonadotropin-releasing hormone by the hypothalamus, which in turn leads to abnormal gonadotropin secretion. There is also a rare disorder characterized by inhibition or irregular menstrual cycles known as primary ovarian insufficiency. Fertility is also influenced by autoimmune diseases, the toxicity of disease-modifying drugs, in particular non-steroidal anti-inflammatory drugs and glucocorticoids. Interestingly, HIV infection is associated with an increased risk of pituitary gland stroke and pituitary lymphoma, possibly leading to hypopituitarism, one of the effects of which is infertility. The pituitary-ovarian axis can also be influenced by thyroid hormones. Hypothyroidism leads to its inhibition, which increases the risk of miscarriage, premature births, stillbirths, and even infertility.

The form of infertility treatment depends on its duration and the patient’s age. It includes ovulation stimulation with clomiphene citrate or gonadotropins, intrauterine insemination, *in vitro* fertilization. Therapeutic regimens are modified and various methods of drug administration are tried to increase their effectiveness and minimize side effects. However, it is important to correctly diagnose the disorder and take the required medications. Obesity is also getting more and more attention, as it is an important factor contributing to the reduction of fertility and predisposing to adverse pregnancy outcomes. Weight loss interventions that include exercise and calorie restriction diets seem to be a good idea. In obese people, metformin is also often used, which improves menstrual cycles, reduces progression from impaired glucose tolerance to type 2 diabetes, and is also recommended in the case of contraindications to the use of hormonal contraceptives. Therefore, infertility as well as disorders of the maintenance of pregnancy and childbirth are a multifactorial issue related to numerous relationships between endocrine organs. Understanding these relationships as well as the importance of the influence of external factors is necessary to develop new and better diagnostic and therapeutic schemes.

## Author contributions

All authors listed have made a substantial, direct, and intellectual contribution to the work and approved it for publication.
